# *Streptococcus agalactiae* glycolipids promote virulence by thwarting immune cell clearance

**DOI:** 10.1126/sciadv.adn7848

**Published:** 2024-05-29

**Authors:** Luke R. Joyce, Sol Kim, Brady L. Spencer, Priya M. Christensen, Kelli L. Palmer, Ziqiang Guan, Julie A. Siegenthaler, Kelly S. Doran

**Affiliations:** ^1^ Department of Immunology and Microbiology, University of Colorado School of Medicine, Aurora, CO, USA.; ^2^ Department of Pediatrics, Section of Developmental Biology, University of Colorado School of Medicine, Aurora, CO, USA.; ^3^ Department of Biological Sciences, The University of Texas at Dallas, Richardson, TX, USA.; ^4^ Department of Biochemistry, Duke University Medical Center, Durham, NC, USA.

## Abstract

*Streptococcus agalactiae* [group B *Streptococcus* (GBS)] is a leading cause of neonatal meningitis, with late-onset disease (LOD) occurring after gastrointestinal tract colonization in infants. Bacterial membrane lipids are essential for host-pathogen interactions, and the functions of glycolipids are yet to be fully elucidated. GBS synthesizes three major glycolipids: glucosyl-diacylglycerol (Glc-DAG), diglucosyl-DAG (Glc_2_-DAG), and lysyl-Glc-DAG (Lys-Glc-DAG). Here, we identify the enzyme, IagB, as responsible for biosynthesis of Glc-DAG, the precursor for the two other glycolipids in GBS. To examine the collective role of glycolipids to GBS virulence, we adapted a murine model of neonatal meningitis to simulate LOD. The GBS∆*iagB* mutant traversed the gut-epithelial barrier comparable to wild type but was severely attenuated in bloodstream survival, resulting in decreased bacterial loads in the brain. The GBS∆*iagB* mutant was more susceptible to neutrophil killing and membrane targeting by host antimicrobial peptides. This work reveals an unexplored function of GBS glycolipids with their ability to protect the bacterial cell from host antimicrobial killing.

## Teaser

*Streptococcus agalactiae* collectively uses its three glycolipids to protect from neutrophil killing by host antimicrobials.

## INTRODUCTION

*Streptococcus agalactiae* [group B *Streptococcus* (GBS)] is a Gram-positive opportunistic pathogen that colonizes the gastrointestinal (GI) tract of healthy adults (*1*) and the lower genital tract of ~30% of women (*2*, *3*). During pregnancy, GBS can be transmitted vertically from a colonized mother to the newborn in utero or during childbirth, which frequently causes early-onset neonatal invasive disease (EOD) such as sepsis and pneumonia in the first week of life (*4*–*7*). GBS is also the leading cause of neonatal meningitis, which can result in mortality rates of up to 9% (*8*) and long-lasting neurological effects in survivors (*4*–*7*). Because of the severity of the resulting diseases, intrapartum antibiotic prophylaxis for colonized pregnant women has been implemented and has drastically reduced the incidence of EOD (*5*, *8*, *9*). However, antibiotic prophylaxis has not altered rates of GBS late-onset disease (LOD), which presents after 1 week of life and accounts for approximately 60% of GBS meningitis cases reported (*10*). Colonization of the neonatal GI tract by bacterial pathogens has been previously observed as a prerequisite for bacteremia and sepsis (*11*, *12*). Thus, GBS LOD is thought to occur following colonization of the neonatal GI tract, which can result from ingestion of infected amniotic fluid in utero, vaginal fluid during childbirth, or infected breast milk postnatally (*8*, *13*–*15*). A recent work has identified that an immature microbiota and an early age promote GBS colonization of the GI tract (*16*). The neonatal gut is more susceptible to translocation of bacterial pathogens due to it being a weaker barrier than in adults because it contains a shorter cell height, lower barrier integrity, as well as a lower innate immune response and underdeveloped immune system (*17*). GBS GI colonization promotes transcriptional changes in the intestinal epithelial barrier resulting in decreased barrier integrity, allowing GBS translocation and systemic spread via the bloodstream (*16*, *18*–*20*). Currently, the only factor known to affect GBS GI colonization is the capsule (*21*), yet other factors that mediate GI colonization, barrier translocation, and systemic spread are unknown.

A dynamic and critical site during host-pathogen interactions is the bacterial cellular membrane. The lipid component of the GBS membrane is primarily composed of anionic phospholipids and neutral glycolipids (*22*–*24*). Little is known about the function and contribution of glycolipids to membrane dynamics in Gram-positive bacteria, although they comprise a large portion of the lipid content. We previously characterized the cellular membrane of GBS and determined that GBS synthesizes three glycolipids: glucosyl-diacylglycerol (Glc-DAG), diglucosyl-DAG (Glc_2_-DAG), and the amino acylated glycolipid, lysyl-Glc-DAG (Lys-Glc-DAG) (*23*–*25*). The addition of glucose to DAG to form Glc-DAG constitutes the first step of GBS glycolipid biosynthesis, although the enzyme responsible for this step has not been identified in GBS. Glc-DAG is subsequently used by two different enzymes to synthesize two independent glycolipids. A second glucose molecule can be added to Glc-DAG to form Glc_2_-DAG, the well-known lipoteichoic acid (LTA) anchor (*25*, *26*), which is catalyzed by the enzyme IagA, invasion-associated gene *A* (*iagA*; GBSCOH1_0636) (*25*). Alternatively, the amino acid lysine is added to Glc-DAG by MprF, multiple peptide resistance factor (*mprF*; GBSCOH1_1931) to form Lys-Glc-DAG (*24*). We have previously investigated the roles of Glc_2_-DAG (GBS∆*iagA*) and Lys-Glc-DAG (GBS∆*mprF*) at the blood-brain barrier (BBB) both in vitro and in vivo using an established murine hematogenous meningitis model (*24*, *25*). We identified roles for Glc_2_-DAG and Lys-Glc-DAG glycolipids in GBS endothelial cell invasion in vitro as well as in pathogenesis of meningitis in vivo, by promoting BBB penetration and brain inflammation.

In this study, we characterized the role of the glycosyltransferase encoding gene *orfB*, which we have named *iagB*, and determined that it encodes the enzyme responsible for biosynthesis of the initial glycolipid, Glc-DAG. We found that GBS∆*iagB* cellular membranes are devoid of all glycolipids yet still synthesize LTA. We identified a unique importance of GBS glycolipids to bloodstream survival during the pathogenesis of meningitis using an established hematogenous murine model of meningitis and a newly adapted neonatal murine model of GBS LOD. We further show that GBS∆*iagB* is more susceptible to neutrophil killing, specifically membrane targeting by antimicrobial peptides (AMPs), which may explain its attenuation in the blood compared to wild-type (WT) GBS. These results greatly increase our knowledge of glycolipid function in GBS and survival mechanisms used against immune cell killing.

## RESULTS

### GBS IagB synthesizes Glc-DAG

**Fig. 1.**
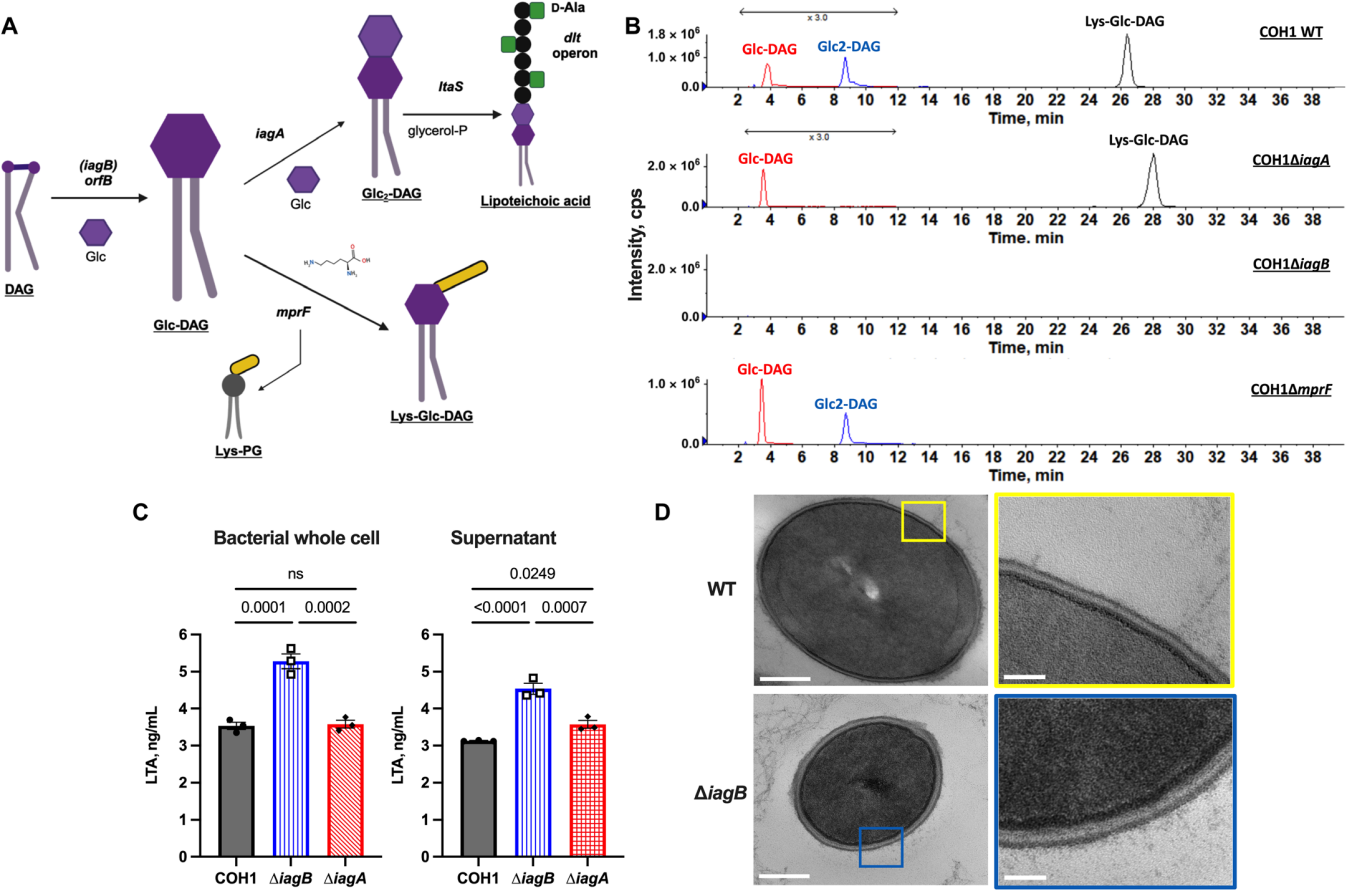
IagB is required for Glc-DAG biosynthesis in GBS. (**A**) Glycolipid biosynthesis pathways in GBS (created in BioRender). (**B**) Major GBS glycolipids (Glc-DAG, Glc_2_-DAG, and Lys-Glc-DAG) are present in COH1 WT. COH1∆*iagA* lacks Glc_2_-DAG, while COH1∆*mprF* lacks Lys-Glc-DAG. As expected, COH1∆*iagB* lacks all three glycolipids, consistent with the fact that IagB is required for the synthesis of Glc-DAG and its derived Glc_2_-DAG and Lys-Glc-DAG. Shown are the positive extracted ion chromatograms. (**C**) ELISA for GBS type I LTA on the bacterial whole cell and culture supernatant. COH1*iagB* synthesizes significantly more LTA and sheds it into the culture supernatant compared to COH1 WT and COH1∆*iagA* [biological and technical triplicate, mean and SEM, ordinary one-way analysis of variance (ANOVA) with Fisher’s least significant difference (LSD)]. *P* values indicated; ns, not significant (*P* value > 0.05). (**D**) Representative TEM images (*n* = 6) of COH1 WT (top) and COH1∆*iagB* (bottom) indicate a thickening of the cell envelope in ∆*iagB* mutant cells. Magnification: left panels, 23k; right panels, 68k. Scale bars: 200 nm (left) and 50 nm (right).

The enzyme responsible for first step of glycolipid biosynthesis [the addition of the first glucose (Glc) to diacylglycerol (DAG) to form Glc-DAG] is uncharacterized. *iagA* is encoded within an operon immediately upstream of the gene *orfB* (GBSCOH1_0637) that contains a glycosyltransferase conserved domain (*25*). We hypothesized that *orfB*, which we now term *iagB*, encodes the enzyme responsible for Glc-DAG biosynthesis (Fig. 1A). To investigate this, we deleted the *iagB* gene in two GBS clinical isolate strains: COH1 (serotype III) (*27*) and CJB111 (serotype V) (*28*). No difference in growth was observed in the COH1 strains, while a minor alteration in growth was observed in the CJB111 background (fig. S1A). Alterations to glycolipid content in the mutant strains were identified by comparing with the WT using normal-phase liquid chromatography (LC) coupled with electrospray ionization mass spectrometry–tandem mass spectrometry (ESI-MS/MS) (Fig. 1B and Table 1). As shown by positive extracted ion chromatograms, we confirmed our previous results that COH1 WT possesses Glc-DAG, Glc_2_-DAG, and Lys-Glc-DAG, COH1∆*iagA* synthesizes Glc-DAG and Lys-Glc-DAG, and COH1∆*mprF* synthesizes Glc-DAG and Glc_2_-DAG (*23*–*25*). Consistent with our hypothesis, COH1∆*iagB* does not synthesize Glc-DAG or the two other glycolipids that are derived from Glc-DAG (Fig. 1B). The absence of glycolipids resulted from the deletion of *iagB* was also confirmed in CJB111 strain background (Table 1 and fig. S1B). The MS analysis for each glycolipid is shown in fig. S1C. These results confirm that IagB is necessary for the synthesis of Glc-DAG and consequently its derived glycolipids.

**Table 1.** Lipidomic profile of GBS strains. GBS lipidomic profiles as characterized in this study and previously published in (*23*, *24*). DAG, diacylglycerol; PG, phosphatidylglycerol; CL, cardiolipin; Lys-PG, lysyl-phosphatidylglycerol; Glc-DAG, glucosyl-diacylglycerol; Glc_2_-DAG, diglucosyl-diacylglycerol; Lys-Glc-DAG, lysyl-glucosyl-diacylglycerol.

	DAG	PG	CL	Lys-PG	Glc-DAG	Glc2-DAG	Lys-Glc-DAG
COH1 WT	**+**	**+**	**+**	**+**	**+**	**+**	**+**
COH1∆*iagB*	**+**	**+**	**+**	**+**	**−**	**−**	**−**
COH1∆*iagA*	**+**	**+**	**+**	**+**	**+**	**−**	**−**
COH1∆*mprF*	**+**	**+**	**+**	**−**	**+**	**+**	**−**
CJB111 WT	**+**	**+**	**+**	**+**	**+**	**+**	**+**
CJB111∆*iagB*	**+**	**+**	**+**	**+**	**−**	**−**	**−**
CJB111∆*iagA*	**+**	**+**	**+**	**+**	**+**	**−**	**−**
CJB111∆*mprF*	**+**	**+**	**+**	**−**	**+**	**+**	**−**

A well-known function of glycolipids, specifically Glc_2_-DAG, in Gram-positive bacteria is to anchor LTA to the membrane (*22*, *25*, *26*, *29*). We hypothesized that GBS∆*iagB* would have altered abundance and increased shedding of LTA, similar to what has been demonstrated previously for GBS∆*iagA* (*25*). LTA abundance was assessed in stationary phase bacteria and culture supernatants of COH1 WT, COH1∆*iagB*, and COH1∆*iagA* via enzyme-linked immunosorbent assay (ELISA) (Fig. 1C). We observed a significant increase in both cell-associated and shed LTA of COH1∆*iagB* compared to COH1 WT, likely due to incorrect anchoring of LTA in the cell membrane. No change in abundance of cell-associated LTA in GBS∆*iagA* was observed, but GBS∆*iagA* shed LTA significantly more compared to COH1 WT, as described previously (*25*). The GBS LTA consists of repeating units of glycerophosphate (GroP) that is modified with an alanine amino acid (*30*). Further analysis of the lipidome of COH1∆*iagB* detected the presence of Ala-2(GroP)-DAG, a repeating unit of LTA, linked to DAG, which is not present in COH1 WT (fig. S1D). This suggests that the loss of the glycolipid anchor in the ∆*iagB* mutant results in the anchoring of LTA to DAG and potentially decreased anchoring strength of DAG leads to increased shedding of LTA.

To further investigate the effects of glycolipid loss on the GBS cell physiology, we performed transmission electron microscopy (TEM) on COH1 WT and COH1∆*iagB*. The ∆*iagB* mutant appears to have a thickened cell envelope compared to WT (Fig. 1D). Furthermore, no differences were observed in the capsule of glycolipid mutants when measured by flow cytometry for the type III capsule of COH1 (fig. S1E). Together, these data indicate that GBS lacking all three glycolipids synthesizes LTA at higher levels, which contains alanine modifications and is anchored to DAG, although less stably.

### IagB contributes to GBS pathogenesis in murine model of hematogenous meningitis

**Fig. 2.**
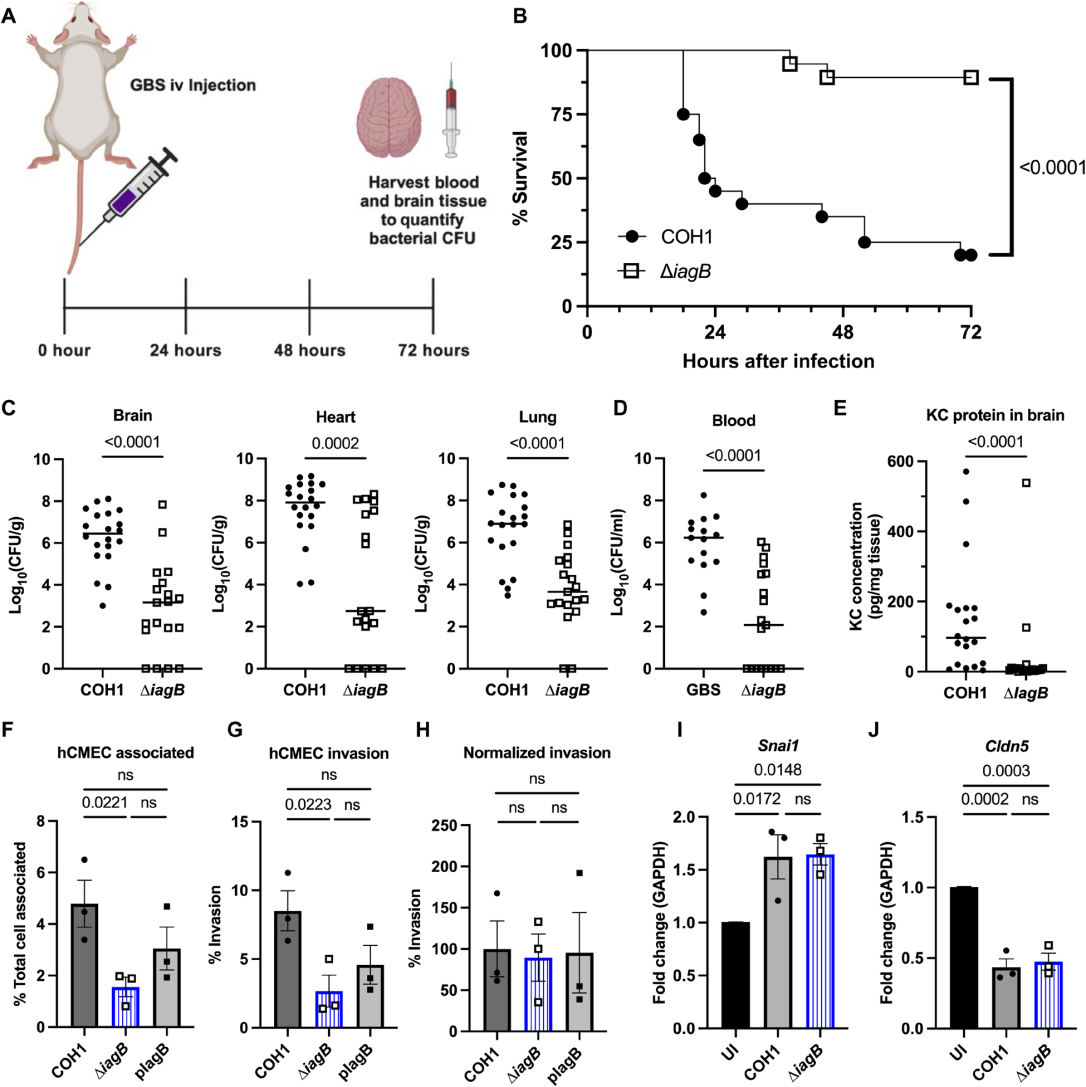
GBS∆*iagB* is attenuated in murine hematogenous meningitis model. (**A**) Schematic of murine hematogenous meningitis model (created in BioRender). (**B**) Kaplan-Meier survival curve of groups of CD-1 mice injected intravenously (iv) with 10^9^ to 10^10^ COH1 WT or COH1Δ*iagB* strains; bacterial counts were assessed in the (**C**) brain, heart, and lungs and (**D**) blood at moribund state or 72 hours (median indicated; WT, *n* = 20; Δ*iagB, n* = 19). (**E**) KC chemokine production measured by ELISA. In vitro assays for (**F**) adherence (total cell associated), (**G**) invasion, and (**H**) normalized hCMEC invasion to total cell associate (WT set to 100%) indicate that *iagB* contributes to adherence but not invasion to brain endothelium (mean of three replicate experiments with four technical replicates, mean and SEM). Transcript levels of (**I**) *Snai1* and (**J**) *Cldn5* in hCMEC cells after 5-hour infection at a multiplicity of infection (MOI) of ~20 (mean of three replicate experiments with three technical replicates, mean and SEM). No difference in transcript levels between WT and ∆*iagB*-infected wells, indicating similar transcriptional changes resulting in BBB permeability. GAPDH, glyceraldehyde-3-phosphate dehydrogenase. Statistical analyses: (B) log-rank test, [(C) to (E)] Mann-Whitney *U* test, and [(F) to (J)] ordinary one-way ANOVA with Fisher’s LSD test. UI, uninfected. *P* values indicated; ns, not significant (*P* value > 0.05).

Previously, we have shown the loss of single glycolipids, either Glc_2_-DAG (∆*iagA* mutant) or Lys-Glc-DAG (∆*mprF* mutant), resulted in diminished ability to invade brain endothelium and penetrate the BBB during meningitis in vivo (*24*, *25*). We hypothesized that GBS∆*iagB*, which lacks all glycolipids, would be similarly attenuated and investigated the contribution of IagB to the development of meningitis using our established model of GBS hematogenous infection (*24*, *25*, *31*). Mice were challenged with either COH1 WT or COH1Δ*iagB* (Fig. 2A). ∆*iagB*-infected mice survived significantly better over 72 hours compared to WT-infected mice (Fig. 2B). To determine bacterial loads in tissues, mice were euthanized either at 72 hours or at a humane endpoint. We recovered significantly less colony-forming units (CFU) in the brain, heart, and lung of Δ*iagB*-infected mice compared to the WT-infected mice (Fig. 2C). Significantly less CFU was recovered from the bloodstream of ∆*iagB*-infected mice compared to WT-infected, indicating that ∆*iagB* is unable to establish bacteremia levels comparable to WT (Fig. 2D). Similar results were observed in mice infected with CJB111 WT and the CJB111∆*iagB* isogeneic mutant, further demonstrating the importance of IagB to GBS pathogenesis (fig. S2, A and B). Furthermore, we examined neutrophil signaling by measuring the neutrophil chemokine KC in brain tissues. WT-infected mice had significantly higher levels of KC protein detected by ELISA compared to ∆*iagB*-infected mice (Fig. 2E). These results establish a role for GBS glycolipids during pathogenesis of meningitis by promoting bloodstream survival.

The decreased bloodstream survival of GBS∆*iagB* results in lower numbers of GBS able to interact with the BBB. To investigate the direct interaction of GBS∆*iagB* with brain endothelial cells, we used the human cerebral microvascular endothelial cell line hCMEC/D3 (Millipore) to mimic the human BBB. In vitro assays for adhesion and invasion were performed as described previously (*24*, *25*, *31*, *32*). There was a significant decrease in the ability of the ∆*iagB* mutant to attach to hCMEC/D3 cells (Fig. 2F) and invade the intracellular compartment (Fig. 2G). However, when the recovered intracellular CFU was normalized to adherent bacteria, there was no difference in the ability of ∆*iagB* to invade the endothelial cells (Fig. 2H). To initiate BBB breakdown, GBS has been shown to cause an increase in expression of the human transcription factor Snail1 (*Snai1*), which results in decreased expression of tight junctions such as Claudin-5 (*Cldn5*), and increased permeability of the BBB (*18*). hCMEC/D3 was infected with COH1 WT and ∆*iagB* for 5 hours, and transcript abundance of *Snai1* and *Cldn5* was analyzed via quantitative polymerase chain reaction (qPCR). We observed the expected increase in *Snai1* and decrease in *Cldn5* transcript levels, but there was no difference between WT and ∆*iagB* infection (Fig. 2, I and J). Together, these data indicate that ∆*iagB* not only is deficient in bloodstream survival but also has a decreased ability to adhere to brain endothelial cells compared to WT; however, once attached to endothelial cells, GBS∆*iagB* is capable of invading and causing transcriptional changes within endothelial cells known to cause BBB barrier permeability similar to GBS WT.

### GBS∆*iagB* is attenuated in a murine model of neonatal meningitis

**Fig. 3.**
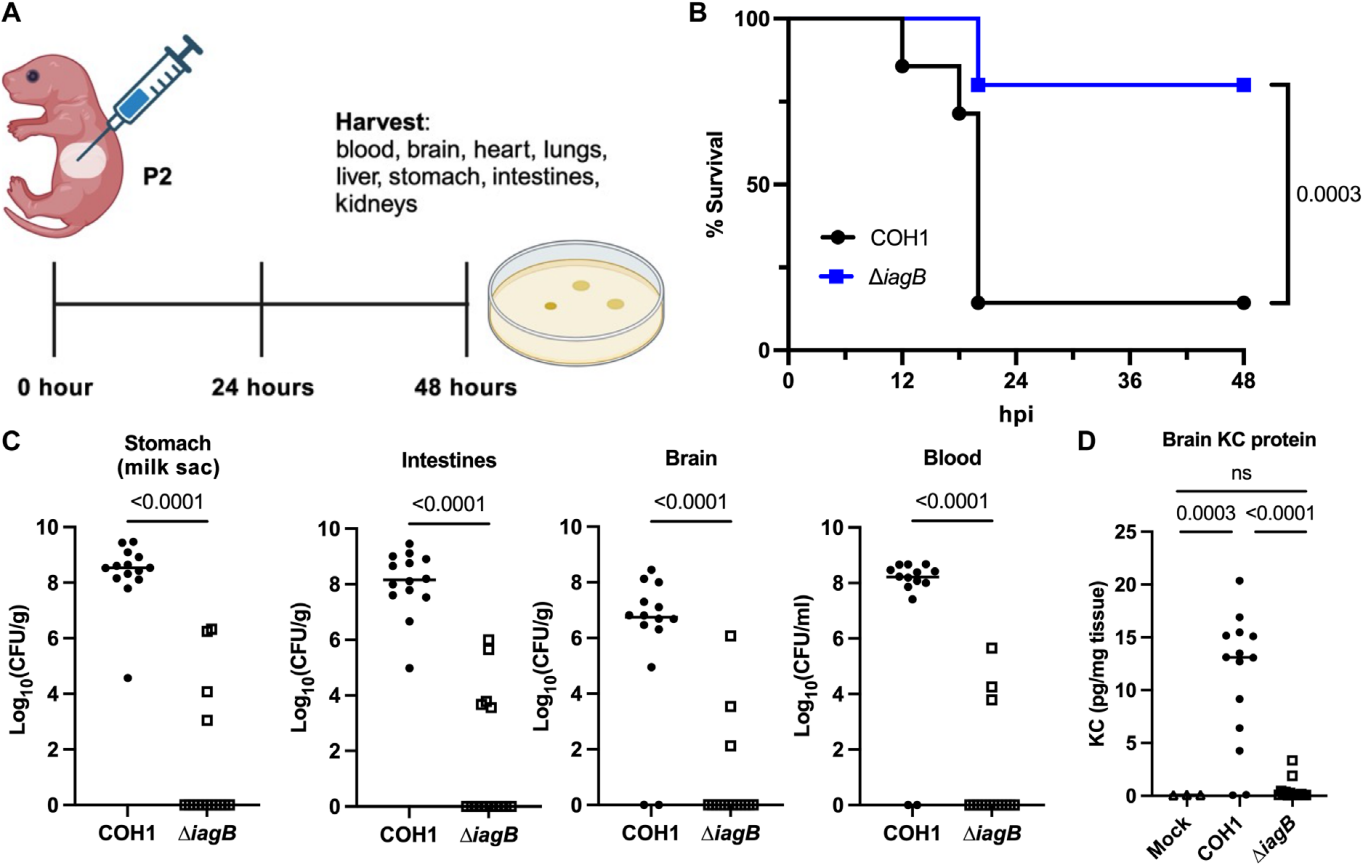
Murine neonatal GBS meningitis model via GI colonization. (**A**) Schematic of murine neonatal GBS meningitis model (created in BioRender). (**B**) Kaplan-Meier survival curve of groups of P2 C57Bl/6J mice injected intragastrically with 10^6^ COH1 WT or COH1Δ*iagB* strains; bacterial counts were assessed in the (**C**) stomach, intestines, brain, and blood at moribund state or 48 hours (median indicated; WT, *n* = 14; Δ*iagB, n* = 15). (**D**) Brain KC chemokine production measured by ELISA. Statistical analyses: (B) log-rank test, (C) Mann-Whitney *U* test, and (D) ordinary one-way ANOVA with Fisher’s LSD test. *P* values indicated; ns, not significant (*P* value > 0.05).

After birth, GBS is capable of colonizing the infant GI tract and can subsequently disrupt the intestinal barrier and spread systemically to the brain, resulting in meningitis and LOD. Previous studies have used murine neonatal models of GBS LOD that use either oral gavage of postnatal day 5 (P5) and P10 pups or intraperitoneal injection of P2 and P5 pups (*16*, *19*–*21*, *33*). We sought to modify the P2 neonatal meningitis infection model to incorporate direct GI colonization of P2 pups to mimic LOD at this age (Fig. 3A). To colonize the gut, a total of 10^6^ CFU of GBS were injected directly into the stomach (milk sac) via intragastric injection of P2 pups and mice were euthanized at 48 hours or at humane endpoint for each mouse, and blood and tissues were collected to determine bacterial burden. Mice infected with COH1∆*iagB* survived significantly better compared to WT-infected mice (Fig. 3B). A significant decrease in recovered COH1∆*iagB* CFU was observed from the stomach, intestines, blood, brain, lung, liver, heart, and kidneys (Fig. 3C and fig. S3A). Results were confirmed using CJB111 WT and CJB111∆*iagB* isogeneic mutant, where we observed a similar significant attenuation of CJB111∆*iagB* in this neonatal infection model (fig. S3, B and C). Next, we examined neutrophil signaling by measuring the neutrophil chemokine KC in brain tissues. A significant increase in KC protein was detected in brains from WT-infected mice compared to uninfected or mice infected with the ∆*iagB* mutant (Fig. 3D). These data indicate that direct colonization of GBS in the GI tract of P2 mice results in systemic spread, BBB penetration, and increased inflammatory signaling in the brains of infected mice. As observed in the hematogenous model of meningitis, IagB also contributes to bloodstream survival and systemic spread during neonatal infection.

### GBS traverses GI epithelial barrier and decreases blood neutrophil abundance

**Fig. 4.**
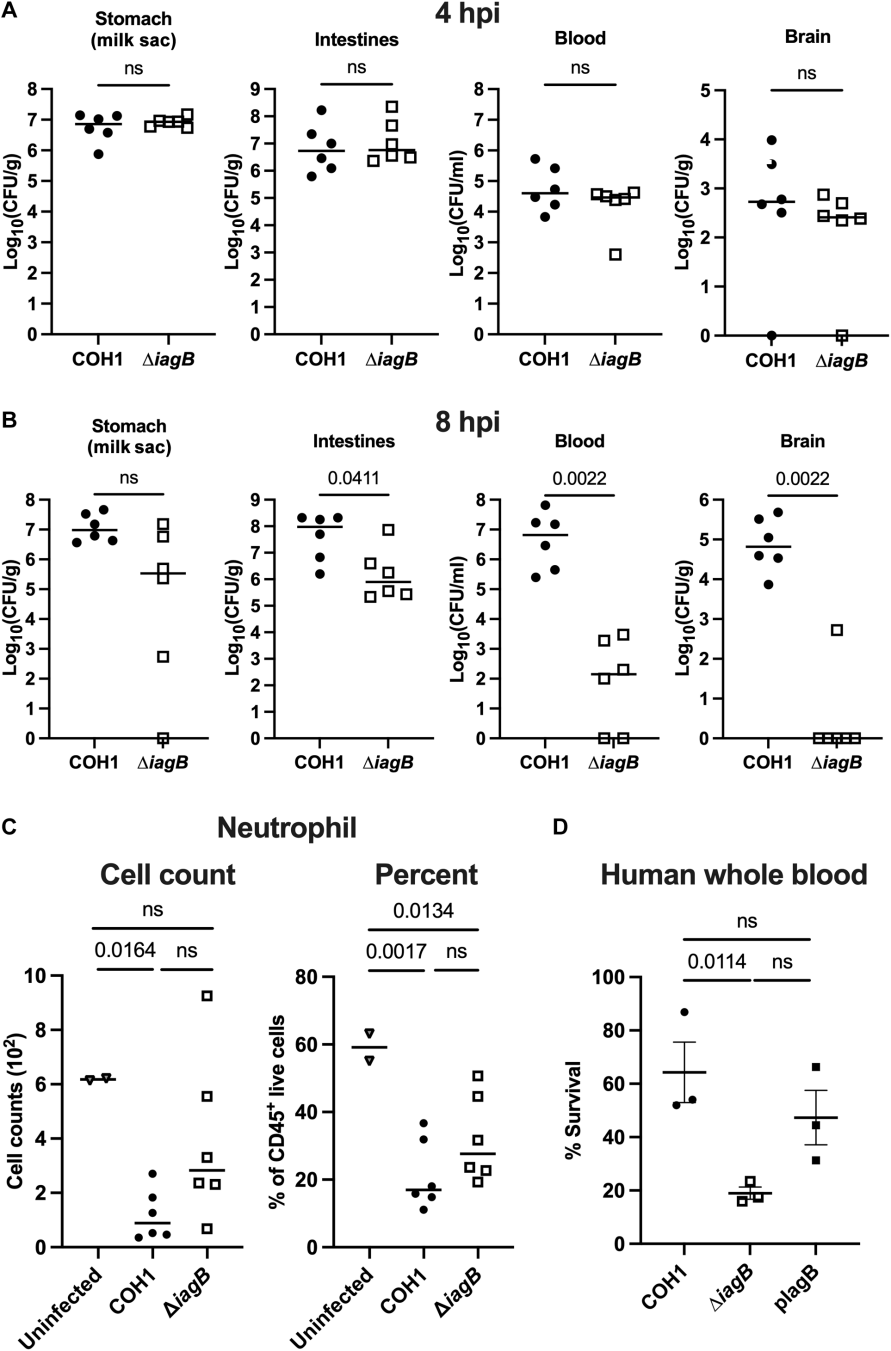
IagB promotes bloodstream survival. Groups of P2 C57Bl/6J mice were inoculated with 10^6^ COH1 WT or COH1∆*iagB* and bacterial burdens assessed at (**A**) 4 hpi and (**B**) 8 hpi. No difference between tissue bacterial burdens was observed at 4 hpi, indicating that GBS∆*iagB* is not defective in traversing the GI epithelial barrier; however, at 8 hpi, significant differences in bacterial burden are observed. (**C**) Blood immune profiling by flow cytometry at 8 hpi of neutrophils. Fewer neutrophils are observed in the blood of WT and ∆*iagB*-infected mice compared to uninfected controls, suggesting that GBS modulates neutrophil numbers during acute infection. (**D**) Pooled human whole blood survival of COH1, COH1∆*iagB*, and complemented strain after 1-hour exposure. COH1∆*iagB* has a significant decrease in survival compared to WT. Percent survival (% Survival) calculated as the ratio of CFU at 1 hour versus input CFU (mean and SEM, biological triplicate). [(A) to (C)] Median indicated. Statistical analyses: [(A) and (B)] Mann-Whitney *U* test and [(C) and (D)] ordinary one-way ANOVA with Fisher’s LSD test. *P* values indicated; ns, not significant (*P* value > 0.05).

In order for GBS to cause disease after GI colonization, it must traverse the GI epithelial barrier (*16*). During neonatal infection, we recovered less of the ∆*iagB* mutant from the GI tract compared to WT at time of euthanasia (Fig. 3C). To investigate if ∆*iagB* is cleared quickly in the GI tract resulting in decreased dissemination or if the mutant crosses the GI epithelial barrier similarly to WT, a timed infection was performed. Mice were infected with 10^6^ WT or ∆*iagB*, and tissues were collected at 4 and 8 hours after infection (hpi). At 4 hpi, no difference was observed between WT and ∆*iagB*-infected mice bacterial burdens in the GI tract, blood, brain, and other tissues (Fig. 4A and fig. S4A). However, at 8 hpi, ∆*iagB*-infected mice exhibited decreased bacterial burden in the GI tract and significantly less CFU burden in the blood, brain, and other tissues compared to COH1 WT-infected mice (Fig. 4B and fig. S4B). This suggests that GBS∆*iagB* is able to traverse the GI epithelial barrier similar to WT within 4 hpi; however, over time, it is more susceptible to clearance.

To characterize the early systemic immune response during GBS neonatal bacteremia, neutrophils and monocytes were quantified in the blood at 8 hpi of WT-infected, ∆*iagB-*infected, and mock-infected mice using flow cytometry (Fig. 4C and fig. S4, C and D). We observed a reduction in the proportion and absolute counts of neutrophils, but not monocytes, in the blood of WT GBS-infected mice compared to mock-infected controls, which may affect GBS clearance. We observed that ∆*iagB*-infected mice exhibited an intermediate phenotype, with higher proportions and absolute blood neutrophil counts compared to WT-infected mice but still trending lower than that of the blood of the mock-infected controls. These data suggest that GBS infection modulates neutrophil numbers in the blood and that this may depend on IagB.

### GBS∆*iagB* does not survive in whole blood in vitro

On the basis of our results thus far, we hypothesized the ∆*iagB* mutant would not survive in whole human blood. We assessed the survival of COH1(pDCErm), COH1∆*iagB*(pDCErm), and COH1∆*iagB*(pIagB) using pooled whole human blood (BioIVT) at 1 hour (Fig. 4D). The ∆*iagB* mutant had a significant decrease in survival compared to WT and the complemented strains. Next, using freshly collected male and female mouse blood, bacterial growth was assessed between empty vector strains COH1(pDCErm), COH1∆*iagB*(pDCErm), and COH1∆*iagB*(pIagB). The COH1∆*iagB*(pDCErm) mutant exhibited decreased growth compared to WT and the complemented strains (fig. S4E). These in vitro data confirm the importance of glycolipids to GBS bloodstream survival.

### GBS∆*iagB* is more susceptible to neutrophil cell killing

**Fig. 5.**
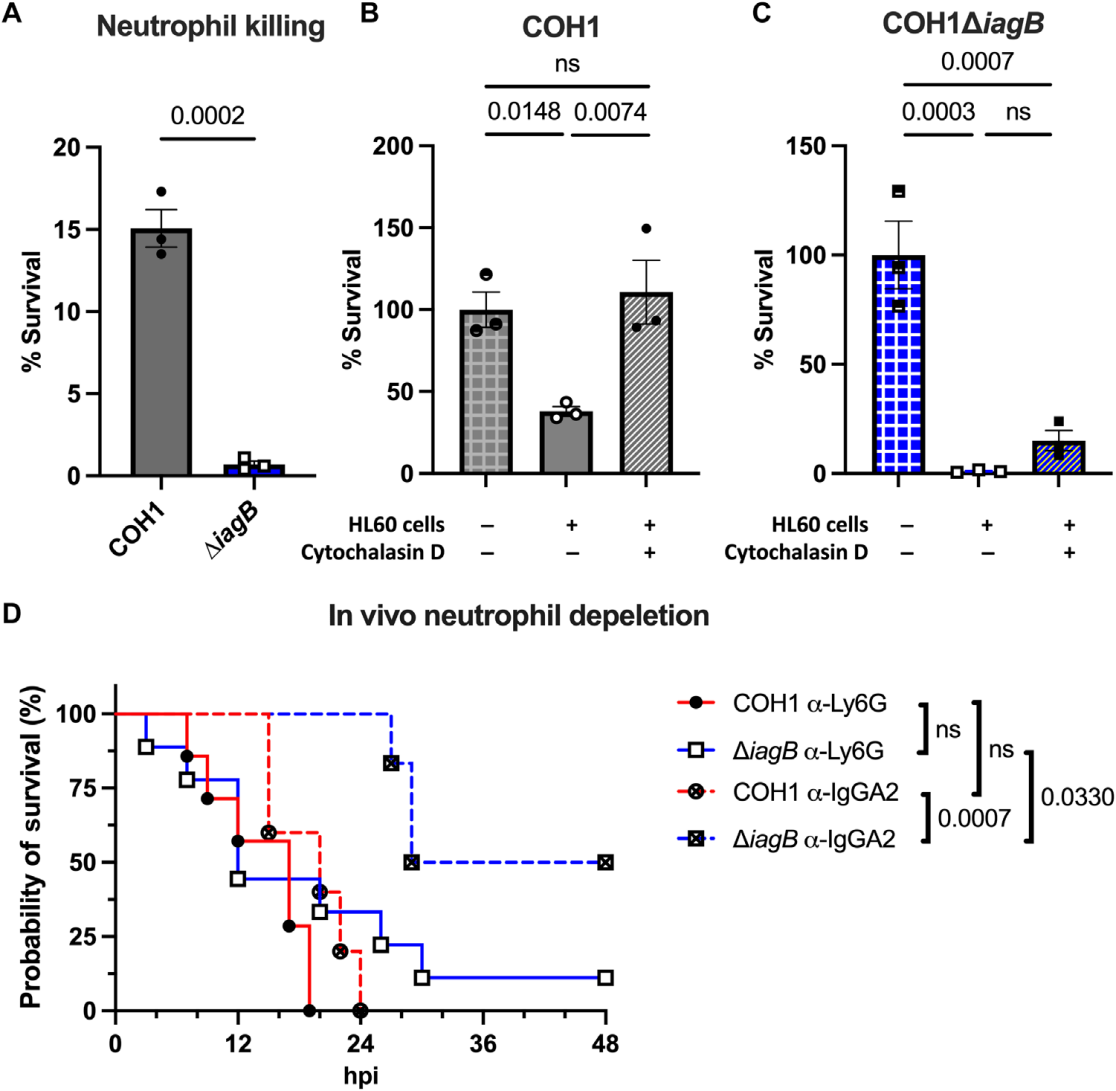
GBS∆*iagB* is more susceptible to neutrophil killing. (**A**) HL60-neutrophil killing of COH1 WT and COH1∆*iagB* at an MOI of 0.01 at 3 hours. % Survival calculated as the ratio of CFU in normal serum versus CFU in heat-killed serum in the presence of HL60 cells. Opsonophagocytic killing of (**B**) COH1 WT and (**C**) COH1∆*iagB*. Cytochalasin D prevents COH1 WT killing but does not completely prevent killing of COH1∆*iagB*, indicating that neutrophils can kill COH1∆*iagB* in a different manner. % Survival calculated as the ratio of CFU in normal serum versus CFU in heat-killed serum and HL60 negative control set to 100% (mean and SEM, biological triplicate). (**D**) Kaplan-Meier curves of neutrophil-depleted (α-Ly6G antibody treated; solid lines) and control (α-IgG2a antibody treated; dashed lines) P2 mice were infected with 10^6^ CFU of COH1 WT (red lines) and COH1∆*iagB* (blue lines). No significant difference observed between WT (red solid line) and ∆*iagB* (blue solid line) survival in neutrophil-depleted mice. ∆*iagB*-infected control mice (blue dashed line) survived significantly better than WT control (red dashed line) and ∆*iagB*-infected neutrophil-depleted (blue solid line) mice. COH1-depleted, *n* = 7; ∆*iagB*-depleted, *n* = 9; WT control, *n* = 5; ∆*iagB* control, *n* = 6. Statistical analyses: (A) unpaired two-tailed *t* test, [(B) and (C)] ordinary one-way ANOVA with Fisher’s LSD test, and (D) log-rank test. *P* values indicated; ns, not significant (*P* value > 0.05).

To investigate if GBS∆*iagB* might also be more susceptible to killing by neutrophils, we used the HL60 [American Type Culture Collection (ATCC) CCL-240] cell line differentiated into neutrophil-like cells in vitro. Exponentially growing GBS was opsonized with 10% normal human serum or heat-killed serum for 15 min prior to the addition of HL60 neutrophils. Incubating COH1 WT or COH1∆*iagB* with HL60 cells for 3 hours resulted in a significant decrease in survival of COH1∆*iagB* compared to COH1 WT (Fig. 5A). No difference in serum killing was observed, and when survival was normalized to HL60 negative control, opsonophagocytic-dependent killing was observed for both strains (Fig. 5, B and C). Using cytochalasin D to block phagocytosis resulted in survival similar of COH1 WT compared to the no HL60 negative control (Fig. 5B). COH1∆*iagB* had improved survival but still a significant decrease in survival compared to the no HL60 negative control (Fig. 5C). Together, these data indicate neutrophils kill GBS in an opsonophagocytic-dependent manner. However, GBS∆*iagB* is more susceptible to both opsonophagocytic and non-opsonophagocytic killing compared to WT, suggesting that neutrophils are able to kill the ∆*iagB* mutant through multiple mechanisms.

### GBS∆*iagB* is not attenuated in neutrophil-depleted neonatal mice

To determine the role of neutrophils during GBS neonatal infection, systemic neutrophil depletion was performed on P1 mice by intraperitoneal injection of α-Ly6G antibody, and nondepleted mice were injected with isotype control α-IgG2a antibody. Confirmation of neutrophil depletion at 24 hours (P2) and 48 hours (P3) after injection was performed using flow cytometry (fig. S5, A and B). P2 neutrophil-depleted and isotype control–treated mice were infected with 10^6^ CFU of WT or ∆*iagB* strains (Fig. 5E). As expected, in the isotype control–treated group, we observed significant mortality in mice infected with WT GBS (red dashed line) compared to the ∆*iagB* mutant (blue dashed line). However, this difference was abrogated in neutrophil-depleted mice as no significant difference in survival was observed between mice infected with WT (red solid line) or ∆*iagB* (blue solid line) strains. Accordingly, within the ∆*iagB*-infected group, we observed a significant decrease in survival between neutrophil-depleted mice (blue solid line) compared to isotype control–treated mice (blue dashed line). Despite large differences in survival, there was no difference in blood CFU between neutrophil-depleted and control mice at time of euthanasia (fig. S5C), suggesting that multiple complex interactions may contribute to attenuation of GBS in the bloodstream. These data provide in vivo confirmation that the presence of neutrophils is important for mouse survival during infection with GBS∆*iagB*.

### GBS glycolipids modulate cellular membrane charge and protect against membrane targeting by AMPs

**Fig. 6.**
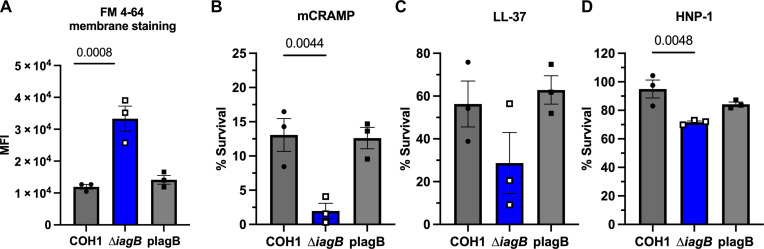
GBS glycolipids protect the membrane from AMPs. (**A**) GBS∆*iagB* has a significantly more negatively charged membrane compared to WT as measured by FM 4-64 membrane staining. GBS∆*iagB* has significantly reduced survival compared to GBS WT after 30 min of exposure to (**B**) mouse mCRAMP (16 μM) and (**C**) human LL-37 (16 μM) and 1 hour exposure to (**D**) human HNP-1 (14.5 μM). Statistical analyses: [(A) to (D)] ordinary one-way ANOVA with Fisher’s LSD test compared to WT. Mean and SEM; *P* values indicated. Biological triplicate with technical replicates performed for each assay.

A primary immune mechanism to combat infection, especially in neutrophils, is the production of cationic AMPs (CAMPs), which are positively charged proteins that target the negatively charged bacterial membrane (*34*, *35*). Because glycolipids are neutrally charged and comprise a large proportion of the bacterial membrane, we hypothesized that the membrane of the GBS∆*iagB* mutant is more negatively charged. To investigate this, we used the membrane stain FM 4-64, which preferentially integrates into negatively charged membranes. Incubation of FM 4-64 with COH1(pDCErm), COH1∆*iagB*(pDCErm), and COH1∆*iagB*(pIagB) resulted in a significant increase in mean fluorescence intensity (MFI) of COH1∆*iagB* compared to COH1 WT and complement strains, indicating a more negatively charged membrane (Fig. 6A). These data suggest that GBS∆*iagB* may be more susceptible to membrane attack by CAMPs.

To investigate if GBS∆*iagB* is more susceptible to CAMPs, we exposed COH1(pDCErm), COH1∆*iagB*(pDCErm), and COH1∆*iagB*(pIagB) to a previously described concentration of mCRAMP, the mouse cathelicidin (*25*, *36*, *37*). We observed a significantly reduced survival of COH1∆*iagB* compared to COH1 WT and complemented strains (Fig. 6B). Furthermore, we confirmed the previous observations that GBS∆*iagA* and GBS∆*mprF* have no change in susceptibility to CAMPs (fig. S6A) (*25*, *37*). Similarly, exposure to the human cathelicidin, LL-37, as well as the human defensin and neutrophil specific peptide, human neutrophil peptide 1 (HNP-1), resulted in decreased survival of COH1∆*iagB* compared to COH1 WT and complemented strains (Fig. 6, C and D). Susceptibility to membrane targeting by CAMPs was confirmed in CJB111(pDCErm), CJB111∆*iagB*(pDCErm), and complement strain CJB111(pIagB) (fig. S6B). We observed similar mortality differences between COH1-infected and COH1∆*iagB*-infected groups (fig. S6C) upon infection of WT mice or mCRAMP knockout (KO) mice (*36*) as well as similar attenuation of growth by the COH1∆*iagB* mutant in blood isolated from mCRAMP KO mice (fig. S6D) compared to that observed previously in blood from WT mice (fig. S4E). These data suggest that multiple host CAMPs, not just mCRAMP alone, may be needed to protect against GBS infection. In addition to increased susceptibility to CAMPs, GBS∆*iagB* exhibited increased susceptibility to membrane-targeting antibiotics daptomycin and colistin as well, but not to cell wall component–targeting antibiotics penicillin and vancomycin (fig. S6E). Also, no difference in susceptibility to hypochlorite or hydrogen peroxide was observed between GBS∆*iagB* and GBS WT strains (fig. S6, F and G). Together, these data indicate that the GBS glycolipids modulate the cellular membrane charge and provide protection against multiple membrane-targeting antimicrobials.

## DISCUSSION

**Fig. 7.**
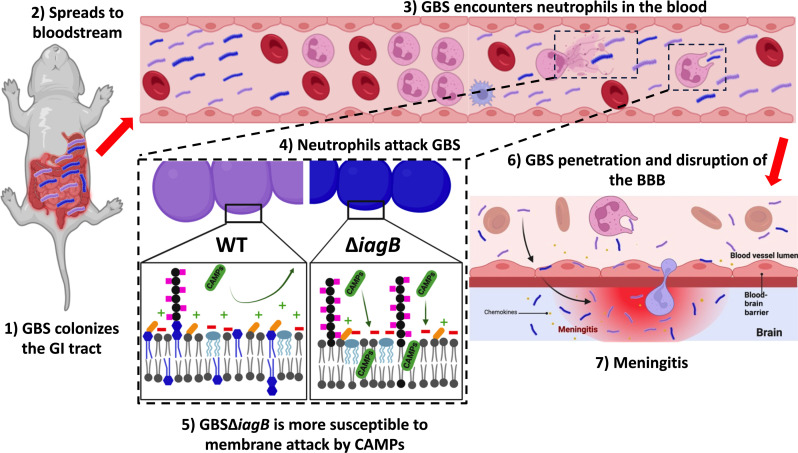
GBS glycolipids promote bloodstream survival. GBS colonizes the neonatal GI tract where it can disrupt the GI epithelial barrier and spread systemically via the bloodstream. Neonatal neutrophils in the bloodstream use CAMPs to control the infection. GBS∆*iagB*, which lacks all glycolipids, is unable to protect the cellular membrane from CAMPs and is cleared at a higher rate than WT GBS. GBS attachment and penetration of the BBB results in BBB disruption and inflammation resulting in meningitis, which is reduced in the absence of glycolipids. Created in BioRender.

In this study, we show that IagB is responsible for the synthesis of the initial glycolipid, Glc-DAG. We found that loss of all the glycolipids in GBS leads to a thickening of the cell envelope as well as altered anchoring and synthesis of LTA. We also determined that GBS glycolipids are important for bloodstream survival in an established hematogenous murine model of meningitis. To further investigate GBS LOD, we adapted a neonatal meningitis model (*16*, *19*, *20*, *33*) by directly seeding the gut of P2 mice with GBS and monitoring systemic spread. In neonatal mice, we observed significant attenuation of GBS∆*iagB* with decreased bloodstream survival compared to WT GBS. The GBS∆*iagB* mutant was not deficient in traversing the neonatal GI epithelial barrier as we show similar CFU burdens in the blood of mice infected with GBS WT or ∆*iagB* mutants at 4 hpi. However, at 8 hpi, significant differences emerge in bloodstream survival and tissue burdens with GBS∆*iagB* decreasing in burden, suggesting increased susceptibility to immune clearance. We further identify a previously unknown role of glycolipids in GBS in protection against neutrophil killing and against membrane targeting by AMPs (Fig. 7).

Aside from the role of Glc_2_-Dag as the LTA lipid anchor, the roles of glycolipids in Gram-positive bacteria remain relatively unknown, although they comprise a large proportion of the cellular membrane (*23*, *24*, *26*). Anchoring of LTA to glycolipids has been shown to be extremely important in staphylococcal physiology as glycolipid mutants display enlarged, misshapen cells and increased shedding of LTA into the environment, likely due to decreased anchoring strength of DAG as the LTA anchor (*26*, *29*, *38*). It has also been observed in enterococci lacking glycolipids that LTA is synthesized, decorated with alanine, and anchored to the cellular membrane via DAG (*39*). Our work similarly shows that GBS devoid of membrane glycolipids produces increased levels of alanine-modified LTA and increased shedding of LTA likely due to the decreased anchoring strength of DAG, as has been observed previously (*25*).

A frontline defense against bacterial pathogens is the production of CAMPs by many different cell types, such as neutrophils, macrophages, and epithelial cells (*40*, *41*). However, neutrophils in particular are relevant immune defenders against extracellular Gram-positive pathogens, particularly in the bloodstream, and newborns rely on neutrophils as the first line of defense against pathogens they encounter (*42*, *43*). Neutrophils in the newborn may have diminished functions compared to adult neutrophils such as decreased granule proteins, lower chemotaxis, and impaired rolling and adhesion, and neonatal pathogens such as GBS are able to cause disease (*42*, *44*–*47*). When neutrophils encounter bacterial pathogens, they use three mechanisms to kill the pathogen: phagocytosis, degranulation, and NETosis, all of which include CAMPs, specifically defensins and cathelicidin (*34*, *35*). CAMPs are positively charged molecules that target negatively charged bacterial membranes. While GBS∆*iagB* did not exhibit increased susceptibility to oxidative stress compared to GBS WT, it was significantly more sensitive to membrane targeting by CAMPs, such as human neutrophil specific peptide HNP-1, an α-defensin, as well as the human and mouse cathelicidin, LL-37 and mCRAMP, respectively. We observed significantly decreased blood neutrophil counts during WT GBS infection, but this decrease was not as notable during GBS∆*iagB* infection. It has been previously observed during *Listeria* infection that neonatal neutrophils are depleted in a Toll-like receptor 2 (TLR2) manner (*43*). Similarly, during GBS neonatal infection, it has been shown that TLR2 induction of interleukin-10 decreases neutrophil recruitment to infected tissues (*33*). We speculate that the decreased numbers of blood neutrophils may promote uncontrolled bloodstream infection by WT GBS, although whether this is similarly mediated by TLR2 signaling remains to be determined. To confirm the importance of neutrophils during GBS bacteremia experimentally, we depleted Ly6G+ cells in neonatal mice and observed that this depletion abrogated our previously observed survival differences during infection between GBS WT and GBS∆*iagB*-infected groups. We observed no difference in bacterial CFU in the blood between neutrophil-depleted and control mice and therefore speculate that altered inflammation during neutrophil depletion may contribute to increased susceptibility to infection with both WT and the ∆*iagB* mutant. It is also possible that increased LTA shedding in the ∆*iagB* mutant may promote increased inflammatory activation, and this warrants further investigation. Together, these data suggest the importance of GBS glycolipids in combating neutrophil defense during GBS neonatal infection.

Bacteria use different strategies to resist the action of host CAMPs. A well-known mechanism is modulation of the bacterial extracellular surface charge. Two primary mechanisms of surface charge modulation are conserved in bacteria: the amino acylation of anionic phospholipids and LTA (*37*, *48*–*50*). Bacterial membranes are primarily negatively charged as they are composed of phosphatidylglycerol (PG) and cardiolipin, both of which are anionic. It was originally found that, in staphylococci, the enzyme, MprF, catalyzes the addition of the positively charged amino acid lysine onto the headgroup of PG to form Lys-PG, thus altering the charge of the membrane and providing resistance to CAMPs (*50*). Our work confirms the previous observation that the GBS MprF does not contribute to CAMP resistance, thus further differentiating GBS’s mechanisms of resistance from that of staphylococci (*37*). Within the streptococci, *mprF*, is not well conserved, and many species have to rely on alternative mechanisms to alter their cellular surface charge (*51*). LTA is a major anionic polymer on the cell surface of Gram-positive bacteria and is essential for survival (*26*). The *dlt* operon confers this positive charge to LTA by decorating the LTA repeating units with d-alanine (*37*, *51*–*54*). Consequently, mutants in the *dlt* operon are more susceptible to CAMPs and decreased in virulence. We observed that GBS∆*iagB* still produces LTA decorated with alanine, indicating that defects in alanylation of LTA are not responsible for phenotypes we observed.

While surface charge is a major bacterial strategy in repelling CAMPs, staphylococci and streptococci have also been shown to use efflux pumps, cell wall density, proteinases to degrade the peptides, and sequestering of the CAMPs by extracellular proteins (*37*, *51*, *52*). In GBS, inactivation of the *dlt* operon by deletion of *dltA* results in in vivo attenuation and increased susceptibility to CAMPs (*37*, *54*). Previous analysis of the cell envelope by TEM of a GBS∆*dltA* mutant revealed a decrease in the cell wall density, and atomic force microscopy revealed a decrease in cell wall rigidity, allowing CAMPs to penetrate through the cell wall easier, rather than a loss of charge repulsion (*37*). Of note, our analysis of the cell envelope in GBS∆*iagB* by TEM reveals no differences in the cell wall density but rather a thickening of the cell envelope. Thickening of the cell envelope has also recently been observed in enterococci upon loss of glycolipids from the membrane (*55*). While our findings suggest that charge repulsion may be a primary mechanism by which GBS glycolipids protect against CAMPs, investigation into CAMP repulsion by these alternative mechanisms warrants further study.

Sustained GBS bacteriemia is required for subsequent BBB penetration and the development of meningitis. During GBS infection, the loss of either glycolipid, Glc_2_-DAG or Lys-Glc-DAG, does not affect bloodstream survival (*24*, *25*), yet the loss of Glc-DAG, which also eliminates both Glc_2_-DAG and Lys-Glc-DAG, resulted in decreased survival in blood in vivo and in the presence of neutrophils in vitro. This may indicate that Glc_2_-DAG and Lys-Glc-DAG have redundant membrane protection functions. Further investigation is also required to determine the individual contribution of each glycolipid to inflammation and BBB disruption. Our results indicate that the loss of all three glycolipids increases the production and shedding of LTA, likely due to unstable anchoring. GBS LTA is a well-characterized TLR2 agonist resulting in acute activation of the proinflammatory response and induction of central nervous system (CNS) injury (*30*, *33*, *56*). It is possible that the increased production and shedding of LTA by GBS∆*iagB* may be hyperinflammatory, trigging a quicker or more robust immune response that results in increased bacterial clearance and may induce BBB disruption and CNS injury. Thus, glycolipids may govern cell wall architecture and dictate the delicate balance of LTA presentation to benefit bacterial survival in the host.

Overall, this work demonstrates the importance of GBS glycolipids to the pathogenesis of meningitis and evasion of immune cell clearance. Neutrophils are a vital component of innate immunity and provide the first line of defense to invading pathogens such as GBS. The ability of GBS to thwart immune cell clearance is critical to the establishment of bacteremia and subsequent brain penetration. The uncharacterized contribution of glycolipids to GBS evasion of neutrophil killing offers an interesting pathway for potential therapeutic targeting, especially for GBS LOD, which is currently not prevented by antibiotic prophylaxis. Future studies aim to elucidate the contribution of GBS glycolipids to the cell surface proteome, barrier breakdown in the GI tract and the BBB, and neonatal-specific neutrophil susceptibilities to GBS infection. This will ultimately provide mechanistic insights into the contribution of glycolipids to GBS disease progression that may have broad implications to other Gram-positive bacterial pathogens.

## MATERIALS AND METHODS

### Bacterial strains, media, and growth conditions

See table S1 for strains used in this study. GBS strains were grown statically at 37°C in Todd-Hewitt broth (THB). *Escherichia coli* strains were grown in lysogeny broth at 37°C with rotation at 225 rpm. Erythromycin and spectinomycin (Sigma-Aldrich) were supplemented to media at 300 and 100 μg/ml for *E. coli* or 5 and 100 μg/ml for GBS strains, respectively.

### Routine molecular biology techniques

All PCR reactions used Phusion polymerase (New England Biolabs). PCR products and restriction digest products were purified using the Qiagen PCR purification kit (Qiagen) per manufacturer’s protocols. See table S2 for primers. Plasmids were extracted using Qiagen plasmid mini and maxiprep kits (Qiagen) per manufacturer’s protocols. Restriction enzyme digests use Xba I, Xho I, Pst I, Bam HI, and Sac II (New England Biolabs) for at least 3 hours at 37°C. Ligations used T4 DNA ligase (New England Biolabs) at 16°C overnight. All plasmid constructs were sequence confirmed by Sanger sequencing (CU Anschutz Molecular Biology Core).

### Construction of KO plasmids

Construction of KO plasmids were performed as described previously (*24*). Briefly, ~1- to 2-kb upstream and downstream of the GBS COH1 *iagB* (GBSCOH1_0637) or CJB111 *iagB* (ID870_05720) and *iagA* (ID870_05725) and the spectinomycin cassette from plasmid pJC303 (*57*) were amplified using PCR. Plasmid, pMBSacB (*58*), and the PCR products were digested using appropriate restriction enzymes and ligated overnight and transformed into chemically competent *E. coli* MC1061 as described previously (*24*). Cultures were pelleted using an Eppendorf 5810R centrifuge at 3900*g* for 10 min at room temperature. Plasmid was extracted as described above, and the insert was PCR amplified and sequence verified via Sanger sequencing (CU Anschutz Molecular Biology Core).

### Generation of electrocompetent GBS cells

Electrocompetent cells were generated as described previously (*24*) with minor modifications. Briefly, a GBS COH1 or CJB111 colony was inoculated in 5 ml of THB + 1.4% or 1.2% glycine, respectively, and grown overnight at 37°C. The 5-ml sample was used to inoculate a second culture of 50-ml prewarmed THB + glycine and grown to an optical density at 600 nm (OD_600nm_) of ~0.3 at 37°C. Cells were pelleted at 3900*g* in an Eppendorf 5810R centrifuge at 4°C for 15 min. Cells were washed once with 25 ml of cold filter-sterilized GBS wash buffer containing 25% PEG-8000 (polyethylene glycol, molecular weight 800) and 10% glycerol in water and pelleted as above. Cell pellets were resuspended in 500 μl of GBS wash buffer and either used immediately for transformation or stored in 75-μl aliquots at −80°C until use.

### Deletion and complementation of GBS COH1 *iagB* and CJB111 *iagB* and *iagA*

The double-crossover homologous recombination KO strategy was performed as described previously (*24*) with the minor modification of spectinomycin (100 μg/ml) was added into THB + 0.75 M sucrose and incubated at 37°C overnight, after the second crossover, before plating on THB agar containing spectinomycin (100 μg/ml). Colonies were PCR screened for loss of genes and grown in the presence of spectinomycin and stocked. Sequence confirmation of the mutants was done via Sanger sequencing (CU Anschutz Molecular Biology Core). Complementation plasmids were constructed as described previously (*24*). Briefly, genes were amplified, restriction digested, and ligated overnight into pDCErm (*59*) plasmid backbone. Ligations were transformed into *E. coli* for maintenance. Complement plasmids were electroporated into GBS electrocompetent cells prepared as described above.

### Lipidomic analyses

Acidic Bligh-Dyer extractions and LC/ESI-MS were performed as described previously (*23*, *24*, *60*, *61*). See Supplementary Text for more details.

### Transmission electron microscopy

TEM was performed at the Electron Microscopy Core Facility at the University of Colorado Anschutz Medical Campus. Approximately 8 ml of mid-exponential (OD_600nm_ of 0.5 to 0.6) growing bacteria were pelleted, and cells were fixed with 2% paraformaldehyde and 2% glutaraldehyde in 0.1 M sodium cacodylate buffer and embedded in 3% agarose. Samples were trimmed into small blocks and rinsed three times in 0.1 M sodium cacodylate buffer, and they were postfixed in 2% osmium tetroxide and 0.8% K_3_[Fe(CN_6_)] in 0.1 M sodium cacodylate buffer for 1 hour at room temperature. Cells were rinsed with water and en bloc stained with 2% aqueous uranyl acetate for 1 hour. They were dehydrated with increasing concentrations of ethanol, infiltrated with SPURR resin, and polymerized in a 60°C oven overnight. Blocks were sectioned with a diamond knife (Diatome) on a UC7 ultramicrotome (Leica) and collected onto copper grids and post-stained with 2% aqueous uranyl acetate and lead citrate. Images were acquired on a Tecnai T12 transmission electron microscope (Thermo Fisher Scientific) equipped with a LaB_6_ source at 120 kV using an NS15 (15 Mpix) camera (AMT).

### Ethics statement

All animal experiments were conducted under the approval of the Institutional Animal Care and Use Committee (no. 00316) at the University of Colorado Anschutz Medical Campus and performed using accepted veterinary standards.

### Murine model of GBS hematogenous meningitis

The murine hematogenous meningitis model was performed as described previously (*18*, *24*, *32*). Briefly, 6- to 8-week-old male CD-1 (Charles River) mice were challenged intravenously with either 1 × 10^9^ to 1 × 10^10 ^ CFU of COH1 WT or COH1Δ*iagB* mutant or 2 × 10^7^ to 3 × 10^7^ CFU of CJB111 or CJB111∆*iagB*. At 72 hpi or moribund state, mice were euthanized and the blood, brain, heart, and lung tissues were harvested, homogenized, and serially diluted on THB agar plates to determine bacterial CFU. For experiments performed in fig. S6C, 10- to 13-week-old female C57BL/6 WT mice (the Jackson Laboratory) and C57BL/6*^Camp−/−^* (mCRAMP KO) mice (*36*) were challenged intravenously with either 1 × 10^9^ CFU of COH1 WT or COH1Δ*iagB* mutant. At 48 hpi or moribund state, mice were euthanized and tissues were harvested, homogenized, and serially diluted on THB agar plates to determine bacterial CFU.

### hCMEC cell adherence and invasion assays

hCMEC/D3 cells (obtained from Millipore) were grown in EndoGRO-MV complete media (Millipore, SCME004) supplemented with 5% fetal bovine serum and fibroblast growth factor 2 (1 ng/ml; Millipore). Cells were grown in tissue culture treated 24-well plates and 5% CO_2_ at 37°C. Assays to determine the total number of bacteria adhered to host cells or intracellular bacteria were performed using GBS empty vector and complement strain as described previously (*24*, *31*, *32*). See Supplementary Text for more details.

### hCMEC *SNAI1* and *CLDN5* qPCR

hCMEC cells were cultured and seeded in 24-well plates as described above. Plates (24 wells) were incubated for 4 days at 37°C and 5% CO_2_. Thirty minutes before infection, media was exchanged for fresh media. Mid-exponential growing COH1 and COH1∆*iagB* were prepared as described above, and hCMEC cells were infected at an MOI of ~20. Infection was performed for 5 hours at 37°C and 5% CO_2_. Cells from each well were collected and RNA extracted using the NucleoSpin RNA purification kit (Macherey-Nagel) per manufacturer’s protocol. cDNA was generated using the Quanta cDNA synthesis kit (Quanta Biosciences), and transcript abundance was determined using PerfeCTa SYBR Green reagent. Fold changes in transcript abundance were calculated using ∆∆CT, by which target gene transcript levels were normalized to those of housekeeping gene, *Gapdh*. Quantitative real-time (qRT) PCR data represent the average of three biological replicates with three technical replicates each. qPCR primers used in this study are listed in table S2.

### Murine neonatal model of GBS meningitis

Male and female P2 C57Bl/6J mice were injected via intragastric injection with 2 × 10^6^ to 3 × 10^6^ CFU of COH1 WT, 1 × 10^5^ CFU of CJB111 WT, the isogenic Δ*iagB* mutants, or phosphate-buffered saline (PBS). Mice were monitored for weight loss, righting reflex, exhaustion levels, and dehydration over 48 hours. At 48 hpi or moribund state, mice were euthanized, and the blood, brain, heart, lung, liver, stomach, intestine, and kidney tissues were harvested, homogenized, and serially diluted on GBS CHROM agar plates to determine bacterial CFU.

### LTA and KC protein ELISA

LTA was detected by ELISA as described previously (*25*). Type I LTA antibody (Novus, clone 55) was used at 1:250 dilution. Overnight bacterial cultures were pelleted and resuspended in THB to an OD_600nm_ of 2, and 100 μl of bacteria or supernatant was added to each well in biological and technical triplicate. KC protein from mouse brain homogenates was detected by ELISA according to the manufacturer’s instructions (R&D Systems).

### Flow cytometry of GBS capsule expression

Flow cytometry for capsule expression was performed using the COH1 capsule-deficient strain HY106, COH1 WT, COH1∆*iagB*, COH1∆*iagA*, and COH1∆*mprF* strains as described previously (*31*) with the slight modification of using HBC buffer [Hank’s balanced salt solution (HBSS)–bovine serum albumin (BSA)–calcium buffer; 1× HBSS without magnesium or calcium, 0.5% bovine serum albumin, and 2.2 mM CaCl_2_] (*62*). Bacteria were washed in HBC, incubated with a purified monoclonal antiserotype III antibody or a monoclonal antiserotype Ia isotype control (provided by J. Kearney, University of Alabama at Birmingham) at a 1:10,000 dilution, washed via centrifugation, and labeled with a donkey antimouse immunoglobulin M conjugated to Alexa Fluor 647 (Invitrogen) at a 1:2000 dilution. All incubations were performed at 4°C with shaking. Stained cells were analyzed on a BD LSRFortessa (BD Biosciences) using the BD FacsDiva software (v9). Data were analyzed with BD FlowJo software version 10.9.

### Flow cytometric analysis of immune cells in neonatal mouse blood

Immune cells were isolated at time of euthanasia from P2 and P3 mouse blood by Ammonium-Chloride-Potassium (ACK) lysis of red blood cells at room temperature (*63*). Cells were washed with RPMI 1640 (Gibco), pelleted at 300*g* for 5 min at 4°C, and resuspended in magnetic-activated cell sorting (MACS) buffer [1× PBS (pH 7.2) + 2 mM EDTA + 0.5% BSA]. Cell suspensions were first stained with eBioscience Fixable Viability Dye eFluor 506 (Thermo Fisher Scientific/Invitrogen, catalog no. 65-0866-18) in PBS for 30 min at room temperature. Cells were stained with the following antimouse surface antibodies in MACS buffer for 30 min at room temperature: F4/80-BV785 (clone BM8; BioLegend, catalog no. 123141); CD45-BUV395 (clone 30-F11; BD Biosciences, catalog no. 564279); CD11b-FITC (clone M1/70; Thermo Fisher Scientific/Invitrogen, catalog no. 11-0112-41) or CD11b-PE (clone M1/70; BD Biosciences, catalog no. 557397); and Ly6G-APC (clone 1A8-Ly6g; Thermo Fisher Scientific/Invitrogen, catalog no. 17-9668-82). After surface antibody staining, the cells were fixed (30 min at room temperature) using the FoxP3 fixation/permeabilization kit (Thermo Fisher Scientific, catalog no. 00-5523-00). Stained cells were analyzed on a BD LSRFortessa (BD Biosciences) using the BD FacsDiva software (v9). Data were analyzed with BD FlowJo software version 10.9.

### Whole human blood killing and mouse blood growth

Pooled human whole blood (BioIVT) killing was performed as described previously (*64*) with minor modifications. Briefly, a total of ~1 × 10^7^ CFU of mid-exponential phase bacteria in 100 μl of PBS were added to 1900 μl of blood. Samples (500-μl aliquots) were incubated at 37°C with end-over-end rotation. At 1 hour, 500 μl of agglutination lysis buffer containing 0.5% saponin (Sigma-Aldrich), 200 U streptokinase K (Sigma-Aldrich), 100 μg of trypsin (Sigma-Aldrich), 2 μg of DNase (Qiagen), and 10 μg of RNase A (Qiagen) in PBS was added and rotated at 37°C for 10 min before serial dilution and plating on THB agar for CFU enumeration. Percent survival was calculated as the ratio of CFU at 1 hour versus input CFU. Whole human blood killing was performed in biological triplicate. Fresh heparinized blood from ~6-week-old male and female C57BL/6J mice was drawn via cardiac puncture. Mid-exponential phase bacteria in PBS were added to blood in Eppendorf tubes at a final concentration of ~1.5 × 10^3^ to 2 × 10^3^ CFU/ml and rotated end over end at 37°C. For CFU quantitation, 10 μl was removed in technical duplicate from each tube and serial diluted and plated on THB agar. Murine blood growth was performed in biological quadruplicate (*n* = 2 male blood and *n* = 2 female blood). mCRAMP-deficient blood growth was performed as described above using fresh heparinized blood from ~6- to 7-week-old male C57BL/6*^Camp−/−^* mice in biological duplicate.

### HL60 opsonophagocytic killing

HL60 cells purchased from ATCC (ATCC CCL-240) were differentiated with 1.25% dimethyl sulfoxide (DMSO) (Sigma-Aldrich) in RPMI for 4 days (*65*). Differentiated HL60 cells were harvested and checked for >65% viability before starting the assay in accordance with a well-characterized protocol (*66*). In a 96-well plate (Corning), 50 μl containing a total of ~1 ×10^3^ CFU of mid-exponential phase GBS, normalized in HBC buffer (1× HBSS, 0.5% BSA, and 2.2 mM CaCl_2_, pH 7.2), were added. Fifty microliters of either 20% normal human serum (Complement Tech) or 20% heat-killed human serum (56°C for >30 min) in HBC buffer was added to each well and incubated at 37°C, with shaking, for 15 min. HL60 cells were preincubated with either 20 μM cytochalasin D (Sigma-Aldrich) or DMSO (Sigma-Aldrich) vehicle control for 15 min, with rotation, before 100 μl of HL60 cells (~1 × 10^5^) was added to each well and incubated at 37°C with shaking (MOI of ~0.01) as described previously (*66*, *67*). After 3 hours, 10 μl was removed, serial diluted, and plated on THB agar for enumeration. Assays were performed in biological triplicate with technical duplicate per replicate. Percent survival was calculated as the ratio of CFU in normal serum versus CFU in heat-killed serum, and no HL60 control wells were set to 100% as described in (*68*).

### CAMP killing

CAMP killing assays were performed with concentrations as described previously (*25*, *36*). A total of 2 x 10^5^ to 4 x 10^5^ CFU of mid-exponential phase cultures were exposed to 16 μM mCRAMP (AnaSpec) in THB and 16 μM human LL-37 (AnaSpec) in 20% THB in PBS for 30 min and 14.5 M HNP-1 in 20% THB in PBS for 60 min in 200 μl at 37°C. Dilutions were plated for CFU enumeration at time points indicated.

### Hypochlorite, hydrogen peroxide, and antibiotic susceptibility

Overnight cultures were diluted 1:100 into 300 μl of THB containing twofold serial dilution of hypochlorite (Clorox), hydrogen peroxide (Sigma-Aldrich), daptomycin (Sigma-Aldrich), colistin (Sigma-Aldrich), penicillin (Sigma-Aldrich), or vancomycin (Sigma-Aldrich) at indicated concentrations in a sterile 96-well plate (Corning). Plates were incubated at 37°C for 24 hours before OD_600nm_ readings were taken on a Tecan Infinite M Plex plate reader.

### Bacterial cell surface charge

FM 4-64 membrane staining was performed on mid-exponential phase cultures growing in THB and normalized to an OD_600nm_ of 0.4 in PBS, and 100 μl was added to a 96-well plate (Corning). Two microliters of FM 4-64 (5 mg/ml; Thermo Fisher Scientific/Invitrogen) was added per well and incubated at 37°C for 20 min before excitation (515 nm) and emission (640 nm) were performed using a Tecan Infinite M Plex plate reader. Readings were first normalized to unstained cells, followed by normalized to PBS with FM 4-64. Assay was performed in biological triplicate with technical duplicate.

### Statistical analyses

All statistics were performed using GraphPad Prism software V10. All relevant statistical analyses are indicated in figure legends.

## Supplementary Materials

### This PDF file includes:

Supplementary Text Figs. S1 to S6 Tables S1 and S2 References
