# Supplementary material for: Streptococcus agalactiae glycolipids promote virulence by thwarting immune cell clearance
Source: Sci Adv. 2024 May 29;10(22):eadn7848. doi: 10.1126/sciadv.adn7848 (PMC11135403; doi:10.1126/sciadv.adn7848)
Supplement: Supplementary file 1 — Supplementary Text Figs. S1 to S6 Tables S1 and S2 References [file sciadv.adn7848_sm.pdf]

Science Advances
AAAS

Supplementary Materials for

Streptococcus agalactiae glycolipids promote virulence by thwarting immune cell clearance

Luke R. Joyce *et al.*

Corresponding author: Kelly S. Doran, kelly.doran@cuanschutz.edu

Sci. Adv. **10**, eadn7848 (2024)
DOI: 10.1126/sciadv.adn7848

This PDF file includes:

Supplementary Text
Figs. S1 to S6
Tables S1 and S2
References

Supplementary Text

Acidic Bligh-Dyer extractions

Centrifugation was performed using a Sorvall RC6+ centrifuge. Cultures were pelleted at 4,280 x *g* for 5 min at room temperature unless otherwise stated. The supernatants were removed, and cell pellets were stored at -80°C until acidic Bligh-Dyer lipid extractions were performed as described (23,24,60). Briefly, cell pellets were resuspended in 1X PBS (Sigma-Aldrich) and transferred to Coring Pyrex glass tubes with PTFR-lined caps (VWR), followed by 1:2 vol:vol chloroform:methanol addition. Single phase extractions were vortexed periodically and incubated at room temperature for 15 minutes before 500 x *g* centrifugation for 10 min. A two-phase Bligh-Dyer was achieved by addition of 100 µL 37% HCl, 1 mL $CHCl_3$, and 900 µl of 1X PBS, which was then vortexed and centrifuged for 5 min at 500 x *g*. The lower phase was removed to a new tube and dried under nitrogen before being stored at -80°C prior to lipidomic analysis.

Liquid Chromatography/Electrospray Ionization Mass Spectrometry

Normal phase LC was performed on an Agilent 1200 quaternary LC system equipped with an Ascentis silica HPLC column (5 µm; 25 cm by 2.1 mm; Sigma-Aldrich) as described previously (23,24,60,61). Briefly, mobile phase A consisted of chloroform-methanol-aqueous ammonium hydroxide (800:195:5, vol/vol), mobile phase B consisted of chloroform-methanol-water-aqueous ammonium hydroxide (600:340:50:5, vol/vol), and mobile phase C consisted of chloroform-methanol-water-aqueous ammonium hydroxide (450:450:95:5, vol/vol). The elution program consisted of the following: 100% mobile phase A was held isocratically for 2 min, then linearly increased to 100% mobile phase B over 14 min, and held at 100% mobile phase B for 11 min. The LC gradient was then changed to 100% mobile phase C over 3 min, held at 100% mobile phase C for 3 min, and, finally, returned to 100% mobile phase A over 0.5 min and held at 100% mobile

phase A for 5 min. The LC eluent (with a total flow rate of 300 µl/min) was introduced into the ESI source of a high-resolution TripleTOF5600 mass spectrometer (Sciex, Framingham, MA). Instrumental settings for negative-ion ESI and MS/MS analysis of lipid species were: IS = -4,500 V, CUR = 20 psi, GSI = 20 psi, DP = -55 V, and FP = -150V. Settings for positive-ion ESI and MS/MS analysis were: IS = +5,000 V, CUR = 20 psi, GSI = 20 psi, DP = +55 V, and FP = +50V. The MS/MS analysis used nitrogen as the collision gas. Data analysis was performed using Analyst TF1.5 software (Sciex, Framingham, MA).

hCMEC cell adherence and invasion assays

Human Cerebral Microvascular Endothelial cells hCMEC/D3 (obtained from Millipore) were grown in EndoGRO-MV complete media (Millipore, SCME004) supplemented with 5% fetal bovine serum (FBS) and 1 ng/ml fibroblast growth factor-2 (FGF-2; Millipore). Cells were grown in tissue culture treated 24 well plates and 5% CO_2 at 37°C. Assays to determine the total number of bacteria adhered to host cells or intracellular bacteria were performed as described previously (24).

Bacteria were grown to mid log phase (OD_{600nm} 0.4-0.5) and normalized to 1 x 10^8 to infect cell monolayers at a multiplicity of infection (MOI) of 1 (1 x 10^5 CFU per well). The total cell-associated GBS were recovered after 30 min incubation. Cells were washed slowly five times with 500 µL 1X PBS (Sigma) and detached by addition of 100 µL of 0.25% trypsin-EDTA solution (Gibco) and incubation for 5 min before lysing the eukaryotic cells with the addition of 400 µL of 0.025% Triton X-100 (Sigma) and vigorous pipetting. The lysates were then serially diluted and plated on THB agar and incubated overnight to determine CFU. Bacterial invasion assays were

performed as described above except infection plates were incubated for 2 h before incubation with 100 µg gentamicin (Sigma) and 5 µg penicillin (Sigma) supplemented media for an additional 2 h to kill all extracellular bacteria, prior to being trypsinized, lysed, and plated as described. Experiments were performed in biological triplicate with four technical replicates per experiment.

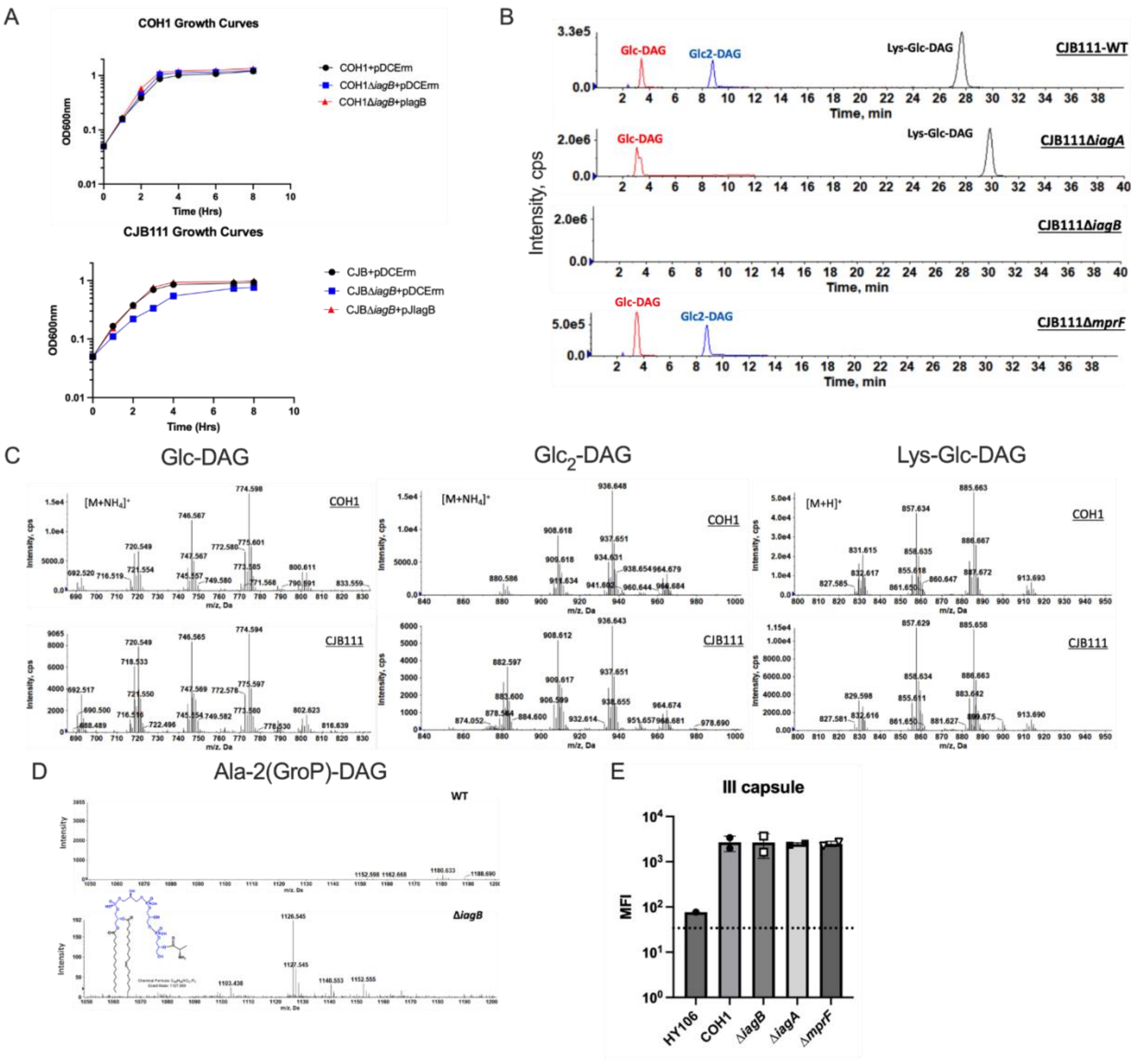

Fig. S1. IagB is necessary for Glc-DAG biosynthesis. A) Growth curves of empty vector strains in THB in COH1 and CJB111 strain backgrounds. No difference in growth is observed in COH1 whereas a growth alteration is observed in CJB111. B) Positive extracted ion chromatograms of Glc-DAG, Glc_2-DAG, and Lys-Glc-DAG in CJB111 WT, CJB111Δ*iagA*, CJB111Δ*iagB*, and CJB111Δ*mprF* strains confirming IagB is required for synthesis of Glc-DAG, the first GBS glycolipid. C) Positive ion mass spectra of the $[M+NH_4]^+$ ion species of Glc-DAG, Glc_2-DAG, and Lys-Glc-DAG in COH1 WT (top panels) and CJB111 WT (bottom panels). D) MS identification of the alanine modified LTA biosynthetic precursor Ala-2(Gro-P)-DAG in COH1Δ*iagB* (lower panel), indicating LTA is anchored to the membrane via DAG. E) Flow cytometry analysis of type III capsule expression in the capsule deficient strain HY106, COH1 WT, COH1Δ*iagB*, COH1Δ*iagA*, and COH1Δ*mprF* strains indicate no difference in capsule expression in glycolipid mutants, performed in biological duplicate. Dashed line represents MFI of isotype control antibody.

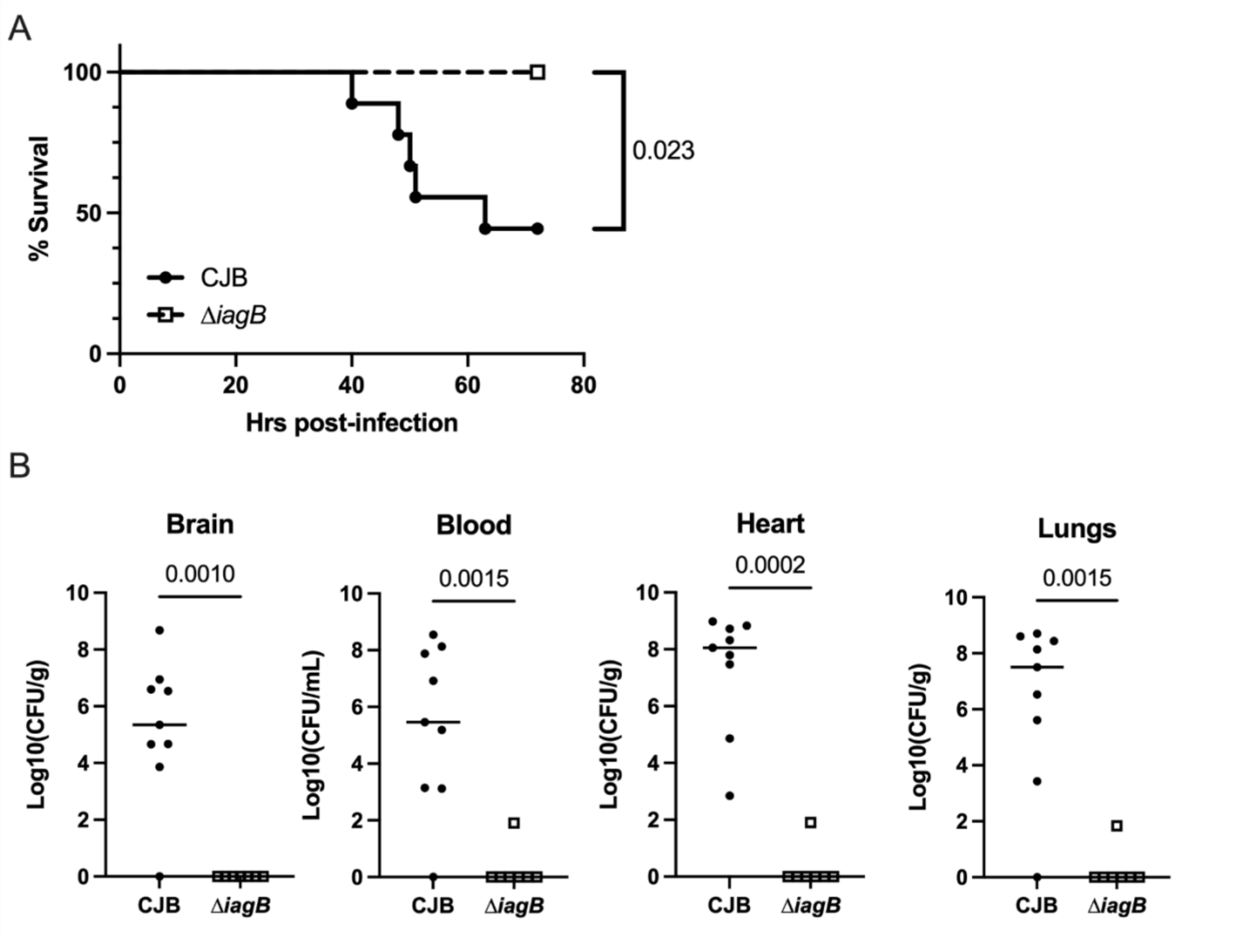

Fig. S2. CJB111Δ*iagB* is attenuated in murine hematogenous meningitis model. A) Kaplan-Meier survival curve of groups of ~ 6-week-old CD-1 male mice were injected intravenously with 107 CJB111 WT or CJB111Δ*iagB* strains and B) bacterial counts were assessed in the brain, heart, lungs and blood at moribund state or 72h, median indicated, WT, n = 9; ΔiagB, n = 7. Statistical tests: A) Log-Rank test and B) Mann-Whitney U tests. P-values indicated.

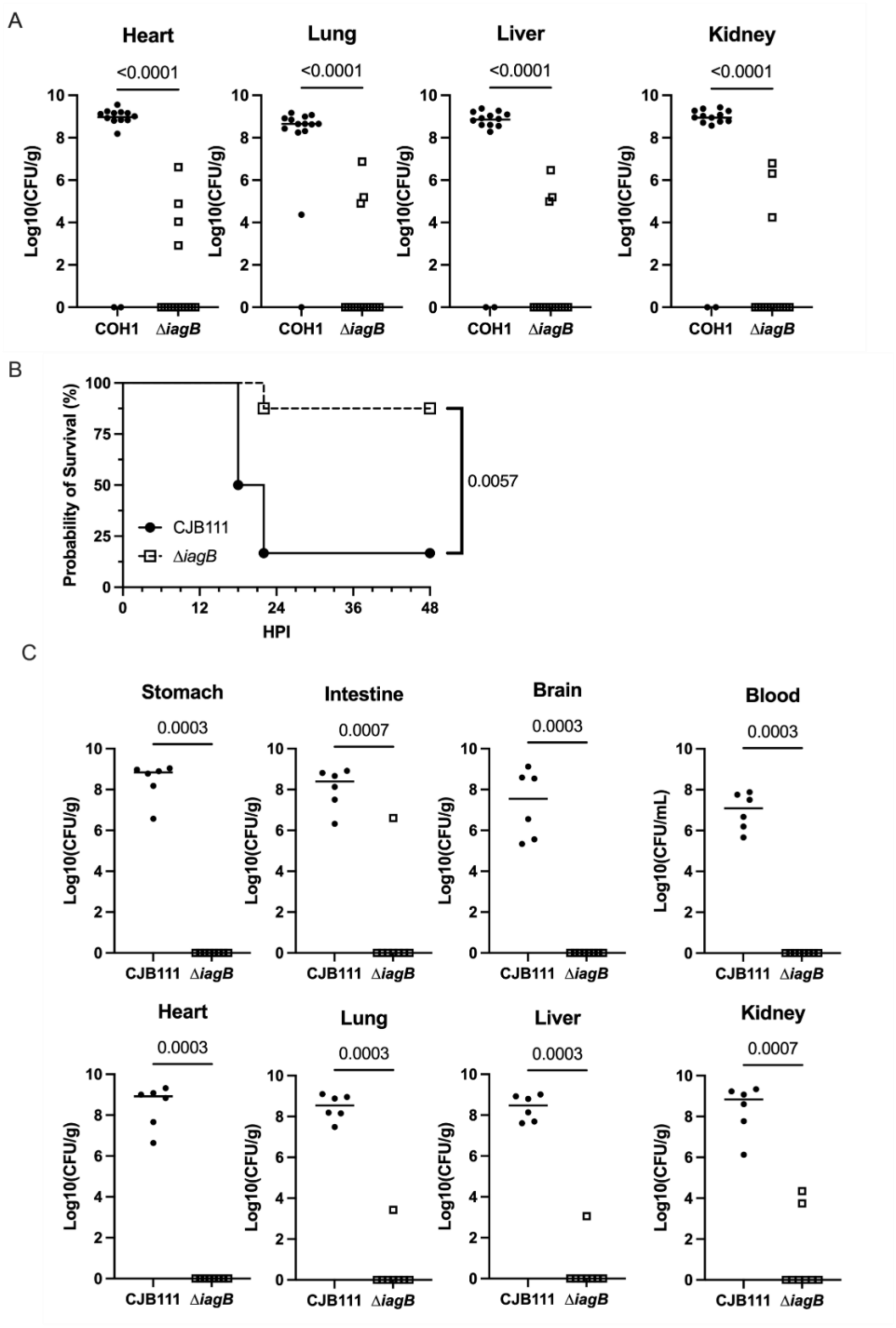

Fig S3. Murine neonatal GBS meningitis model. A) Bacterial burdens of COH1 WT and COH1Δ*iagB* in heart, lung, liver, and kidney. B) Kaplan-Meier curve of P2 neonatal mice infected with ~1 x 10^5 CFU of CJB111 WT or CJB111Δ*iagB* (WT; n = 6, Δ*iagB*; n = 8) indicates CJB111Δ*iagB* infected mice survive significantly better than WT infected mice and C) significantly reduced bacterial burdens recovered in tissues of mice infected with CJB111Δ*iagB* compared to CJB111 WT. Statistical analyses: A,C) Mann-Whitney U test, B) Log-Rank test. P-values indicated.

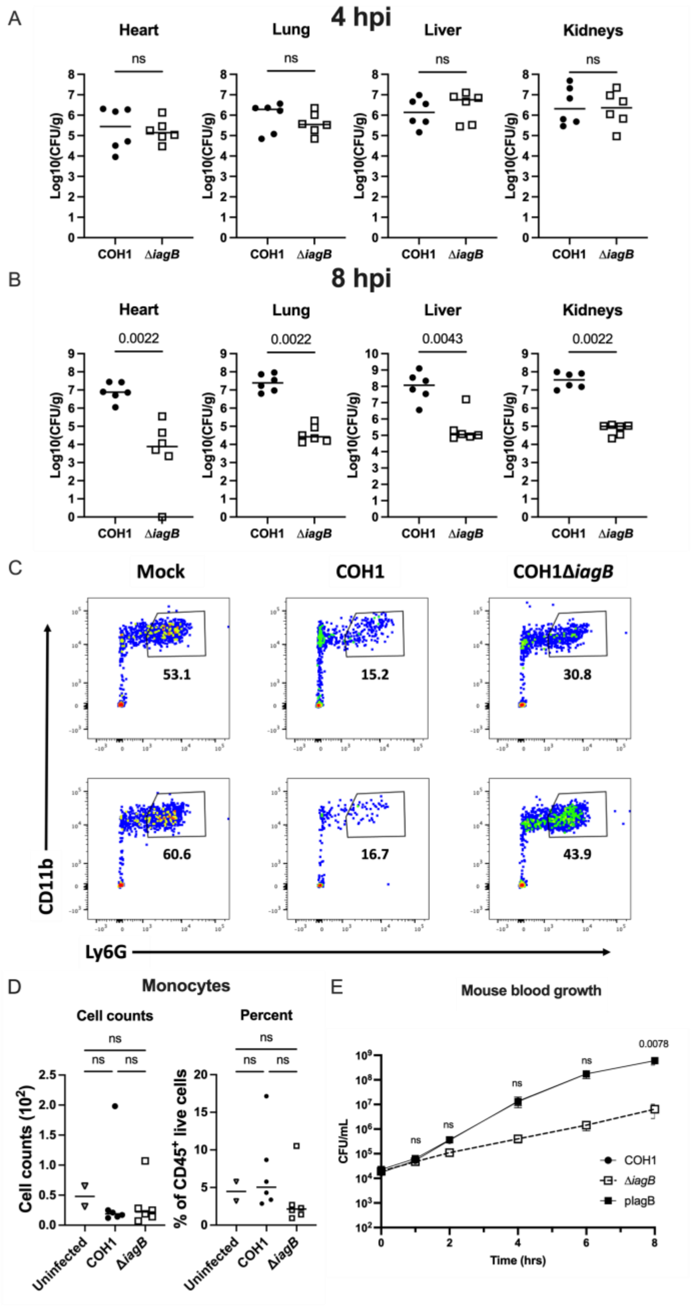

Fig S4. Bacterial burden at 4 and 8 hpi and representative flow plots showing blood neutrophils. Bacterial burden in neonatal tissues at A) 4 hpi and B) 8 hpi. C) Representative flow plots of neutrophil staining, two representative plots shown per infection group. D) Blood immune profiling by flow cytometry at 8 hpi of monocytes, median indicated. E) Growth in murine whole blood indicates Δ*iagB* does not grow as well compared to WT and complemented strains. Mean and SD, biological quadruplicate. Statistical analyses: A-B) Mann-Whitney U tests. D) Ordinary One-Way ANOVA with Fishers LSD test. E) Two-Way ANOVA with Fisher's LSD test, p-values indicated are between WT and Δ*iagB*. P-values indicated, ns; not significant (p-value > 0.05).

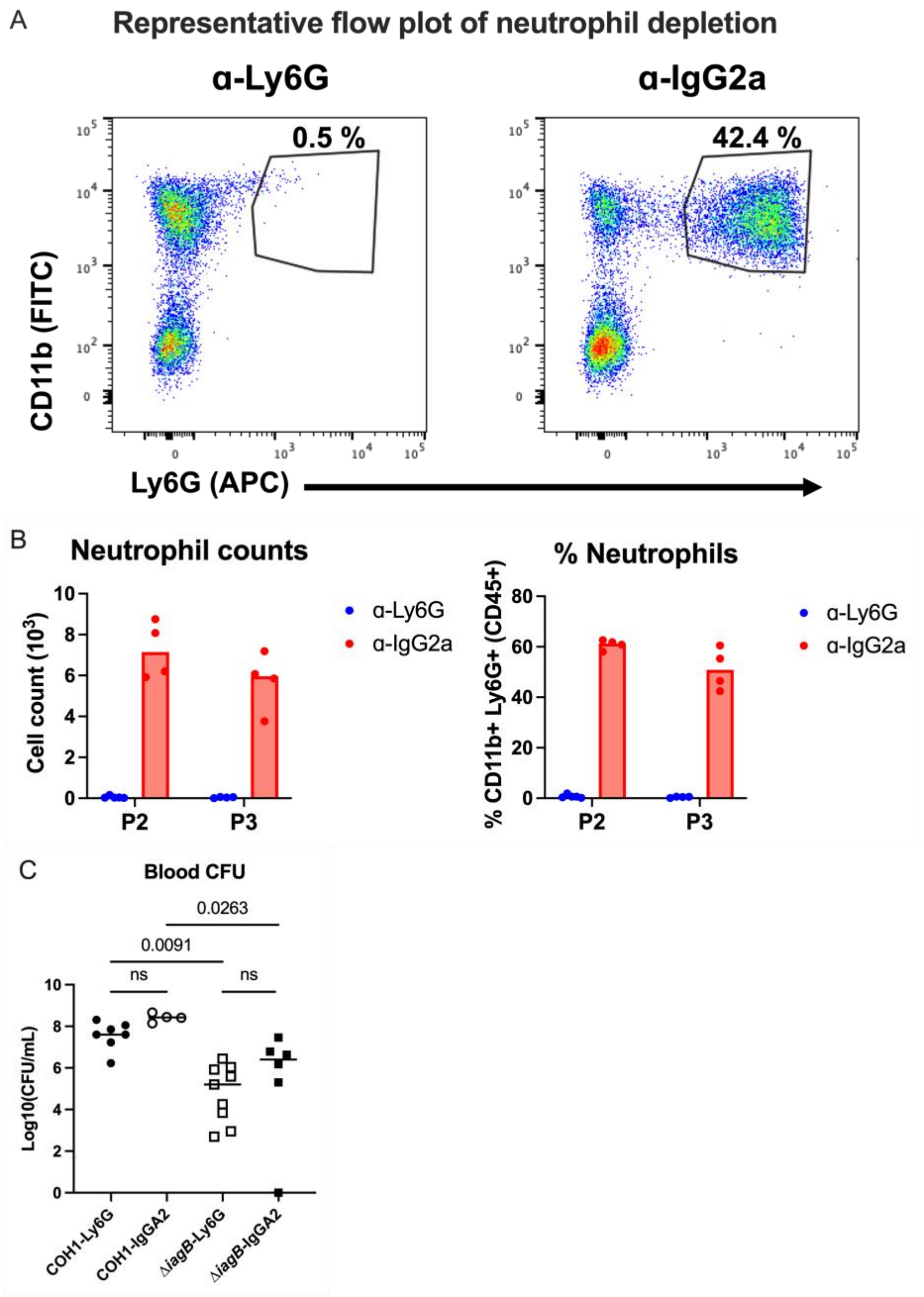

Fig S5. Confirmation of neutrophil depletion from blood of neonatal mice. A) Representative flow plots of $Ly6G^+$ cell detection. B) Cell counts and percentage of live CD45+ cells in α-Ly6G (n = 9) and α-IgG2a isotype control (n = 8) injected mice at 24h (P2) and 48h (P3) post injection. C) CFU recovered of COH1 WT and COH1Δ*iagB* in the blood of neutrophil depleted (Ly6G) mice and isotype control (IgGA2) mice at time of sacrifice. Median, Kruskal-Wallis test. P-values indicated, ns; not significant (p-value > 0.05).

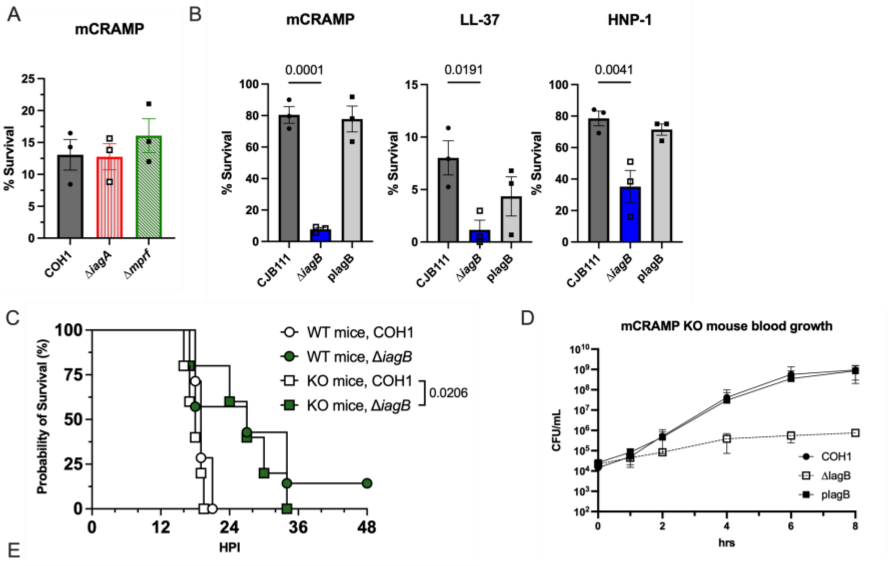

E

Minimum inhibitory concentrations

Strain	Penicillin (µg/mL)	Vancomycin (µg/mL)	Daptomycin (µg/mL)	Colistin (µg/mL)
COH1	0.125	1	8	64
COH1Δ*iagB*	0.125	1	2	32
COH1Δ*iagB*::pIagB	0.125	1	8	64
COH1Δ*iagA*	0.125	1	4	64
COH1Δ*iagA*::pIagA	0.125	1	8	64
COH1Δ*mprF*	0.125	1	4	64
COH1Δ*mprF*::pMprF	0.125	1	8	64
CJB111	0.0625	0.5	8	128
CJB111Δ*iagB*	0.0625	0.5	2	64
CJB111Δ*iagB*::pIagB	0.0625	0.5	8	128
CJB111Δ*iagA*	0.0625	0.5	4	128
CJB111Δ*iagA*::pIagA	0.0625	0.5	8	128
CJB111Δ*mprF*	0.0625	0.5	4	128
CJB111Δ*mprF*::pMprF	0.0625	0.5	8	128

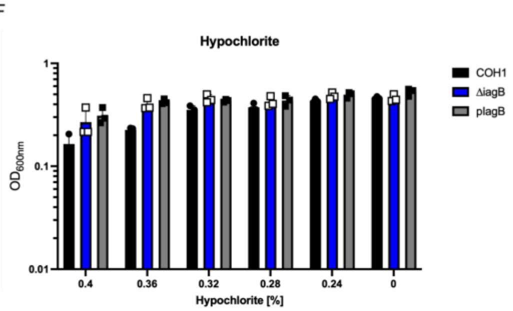

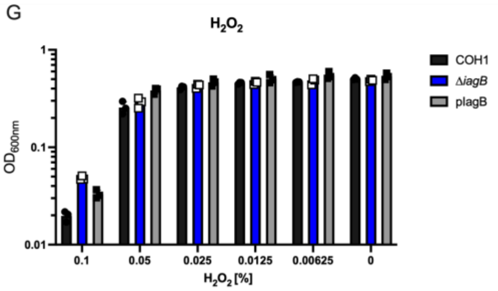

Fig S6. GBSΔ*iagB* is more susceptible to membrane targeting antimicrobials. A) No difference in mCRAMP (16 µM) susceptibility between COH1 WT, COH1Δ*iagA* and COH1Δ*mprF* strains, as published previously. B) CJB111 susceptibility to mCRAMP (16 µM) and human LL-37 (16 µM) at 30min, and human HNP-1 (14.5 µM) at 1h. Mean and SEM. C) Kaplan-Meier survival curve of C57BL/6 WT (circles) and mCRAMP KO mice (squares) infected with injected intravenously with 10^9 COH1 WT (white symbols) or COH1Δ*iagB* (green symbols) strains for 48 hpi (WT mice - COH1; *n* = 7, Δ*iagB*; *n* = 7, mCRAMP KO mice – COH1; *n* = 5, Δ*iagB*; *n* = 5). D) Growth in mCRAMP deficient murine whole blood indicates Δ*iagB* does not grow as well compared to WT and complemented strains. Mean and SD, biological duplicate. E) Minimum inhibitory concentrations of glycolipid mutants to Daptomycin, Colistin, Penicillin, and Vancomycin in THB. No difference is observed between COH1(pDCErm), COH1Δ*iagB*(pDCErm), and COH1Δ*iagB*(pIagB) susceptibility to F) hypochlorite and G) hydrogen peroxide, mean and SD. Statistical analyses: A,B) Ordinary One-way ANOVA with Fishers LSD test compared to WT, C) Log-Rank test. P-values indicated. All assays performed in biological triplicate unless stated.

Supplemental Table S1. Strains and plasmids used in this study.

Organism	Strain	Description	Ref
	COH1	Wild-type *S. agalactiae* strain, serotype III	(27)
	COH1Δ*iagB*	*iagB* (GBSCOH1_0637) deletion strain	This work
	COH1Δ*iagB(*pDCErm)	Empty vector control strain	This work
	COH1Δ*iagB*(pIagB)	Expresses COH1 *iagB* in pDCErm	This work
	COH1(pDCErm)	Empty vector control	This work
	COH1Δ*iagA*	*iagA* (GBSCOH1_0636) deletion strain	(25)
	COH1Δ*iagA(*pDCErm)	Empty vector control	(25)
	COH1Δ*iagA*(pIagA)	Expresses COH1 *iagA* in pDCErm	(25)
	COH1Δ*mprF*	*mprF* (GBSCOH1_1931) deletion strain	(24)
	COH1Δ*mprF(*pDCErm)	Empty vector control	This work
	COH1Δ*mprF*(pCMprF)	Expresses COH1 *mprF* in pDCErm	This work
	HY106	COH1Δ*cpsD*	(69)
	CJB111	Wild-type *S. agalactiae* strain, serotype V	(70,71)
	CJB111Δ*iagB*	*iagB* (ID870_05720) deletion strain	This work
	CJB111Δ*iagB*(pDCErm)	Empty vector control strain	This work
	CJB111Δ *iagB*(pJIagB)	Expresses CJB111 *iagB* in pDCErm	This work
	CJB111Δ*iagA*	*iagA* (ID870_05725) deletion strain	This work
S. agalactiae	CJB111Δ*iagA*(pDCErm)	Empty vector control strain	This work
	CJB111Δ *iagB*(pJIagA)	Expresses CJB111 *iagA* in pDCErm	This work
	CJB111Δ*mprF*	*mprF* (ID870_10050) deletion strain	(24)
	CJB111Δ*mprF*(pDCErm)	Empty vector control strain	(24)
	CJB111Δ*mprF*(pJMprF)	Expresses CJB111 *mprF* in pDCErm	(24)
	CJB111(pDCErm)	Empty vector control strain	(24)
	MC1061	Plasmid cloning host; F^-, araD139, Δ(araABC-leu)7696, Δ(lac)X74, galU, galK, hsdR2, ($r_K^- m_K^+$), mcrB1, rpsL, (Str^r)	(72)
	MC1061(pDCErm)	Empty vector control	(24)
	MC1061(pIagB)	Expresses COH1 *iagB* from $P_{tetM/erm}$ in pDCErm	This work
	MC1061(pCMprF)	Expresses COH1 *mprF* from $P_{tetM/erm}$ in pDCErm	This work
	MC1061(pJIagB)	Expresses CJB111 *iagB* from $P_{tetM/erm}$ in pDCErm	This work
	MC1061(pJIagA)	Expresses CJB111 *iagA* from $P_{tetM/erm}$ in pDCErm	This work
	MC1061(pJMprF)	Expresses CJB11 *mprF* from $P_{tetM/erm}$ in pDCErm	(24)
	MC1061(pCIagBKO)	Allelic exchange plasmid containing ~2 kb sequence flanking GBSCOH1_0637	This work
	MC1061(pJIagBKO)	Allelic exchange plasmid containing ~2 kb sequence flanking ID870_05720	This work
	MC1061(pJIagAKO)	Allelic exchange plasmid containing ~2 kb sequence flanking ID870_05725	This work

Plasmid	Description	Ref
pDCErm	Constitutive expression vector for streptococcus from $P_{tetM/erm}$	(57)
pIagB	pDCErm expressing COH1 *iagB* (GBSCOH1_0637)	This work
pCMprF	pDCErm expressing COH1 *mprF* (GBSCOH1_1931)	This work
pJIagB	pDCErm expressing CJB111 *iagB* (ID870_05720)	This work
pJIagA	pDCErm expressing CJB111 *iagA* (ID870_05725)	This work
pJMprF	pDCErm expressing CJB111 *mprF* (ID870_10050)	(24)
pMBSacB	Allelic exchange plasmid for *S. agalactiae*. Confers erythromycin resistance and sucrose sensitivity	(58)

pCIagBKO	Knockout plasmid containing ~2 kb sequence flanking GBSCOH1_0637	This work
pJIagBKO	Knockout plasmid containing ~2 kb sequence flanking ID870_05720	This work
pJIagBKO	Knockout plasmid containing ~2 kb sequence flanking ID870_05725	This work
pJC303	Used for amplification of Spectinomycin resistance gene	(27)

Supplemental Table S2. Primers used in this study.

Primer	5' – 3' sequence	Use
pF	AGCGCTAGGAGGAAAC	For pDCErm plasmid insert sequencing
pR	CCCATGCCATCTCCAATC	For pDCErm plasmid insert sequencing
**GBSCOH1_0637, ID870_05720, ID870_05725 knockout plasmid construction, sequencing, and integration screening**		
IagBup_F_XhoI	ACGTCACTCGAGATGAAAGTTTTACTGT ATTTAG	5' most primer of upstream fragment, amplifies with BupR_PstI
IagBupR_PstI	ACGTCACTGCAGCTTACAACTCCATTAC TTTTTG	3' most primer of upstream fragment, amplifies with BupF_XhoI
IagBBDwnF_BamHI	ACGTCAGGATCCGACGAATAAAATTAGT AAATGTC	5' most primer of downstream fragment, amplifies with BDwn_R_NotI
IagBDwn_R_NotI	ACGTCAGCGGCCGCTACCACGCCAGTA AGC	3' most primer of downstream fragment, amplifies with BDwnF_BamHI
IagBS1	CCACACGATAACCTTC	Sequencing primer with BOutF/T7
IagBS2	GTTGAATTAGCGACTGATG	Sequencing primer with SpecS1
IagBS3	GTTGAATTAGCGACTGATG	Sequencing primer with SpecS2
IagBS4	GAAAGAGTGCAATTAGAGAAC	Sequencing primer with BOutR/T3
IagBOutF	GTCTATCTCGGCTTGGGTC	Anneals 5' outside integration arms
IagBOutR	CAAACCAAGCAGTACCATAG	Anneals 3' outside integration arms
IagBF_SacII	ACGTCACCGCGGATGCGTATAGGTCTAT TTAC	Amplifies GBSCOH1_0637/ID870_05720 with BR_BamHI
IagBR_BamHI	ACGTCAGGATCCTCAATCTAAAAAATCTC GAATAG	Amplifies GBSCOH1_0637/ID870_05720 with BF_SacII
Up_IagAF_XhoI	ACGTCACTCGAGTTAGAGGTGTAATATG ACAAATG	5' most primer of upstream fragment, amplifies with UpIR_PstI
Up_IagAR_PstI	ACGTCACTGCAGAGGTCTTATTATATCTC ATTTTACC	3' most primer of upstream fragment, amplifies with Up_I_F_XhoI
Dwn_IagAR_NotI	ACGTCAGCGGCCGCCGTCTCAATCTAAA AAATCTC	3' most primer of downstream fragment, amplifies with DwnIF_BamHI
DwnIagAF_BamHI	ACGTCAGGATCCGATGCGTATAGGTCTA TTTAC	5' most primer of downstream fragment, amplifies with Dwn_IR_NotI
IagA_S1	CAAAAAAATTCATATGC	Sequencing primer with Out_IF/T7
IagA_S2	CATTAATGATATTCGTAC	Sequencing primer with SpecS1
IagA_S3	TGACACCATCCAAATC	Sequencing primer with SpecS2
IagA_S4	GGCAAATTGATTAAGC	Sequencing primer with Out_IR/T3
Out_IagAF	ACATGATATATTGAGAGG	Anneals 5' outside integration arms
Out_IagAR	ACCTAACATGTCTTC	Anneals 3' outside integration arms
SpecF_PstI	ACGTCACTGCAGGTGAGGAGGATATATT TG	Amplifies Spec cassette
SpecR_BamHI	ACGTCAGGATCCTTATAATTTTTTTAATC TGT	Amplifies Spec cassette
IagAF_SacII	ACGTCACCGCGGATGAAAGTTTTACTGT ATTTAG	Amplifies ID870_05725 with IagR_BamHI
IagAR_BamHI	ACGTCAGGATCCTTACAACTCCATTACTT TTTG	Amplifies ID870_05725 with IagF_BamHI
T7 promoter	TAATACGACTCACTATAGGG	Amplifies with MpS5F below to sequence plasmid, amplifies with T3 promoter for insert screening and plasmid presence
T3 promoter	AATTAACCCTCACTAAAGGG	Amplifies with MpS3R below, amplifies with T7 promoter for insert screening and plasmid presence
SpecS1	CACTATTTGGTTTTAGTCCACTC	Anneals in Spec cassette
SpecS2	GTATGATTTTAACTATGGACACG	Anneals in Spec cassette
**qPCR primers**		
SNAIL1 rev	ATTCGGGAGAAGGTCCGAGC	(18)
SNAIL1 For	GGACCCACACTGGCGAGAAG	(18)
hClaudin5 Rev	CACAGACGGGTCGTAAAACTC	(18)
hClaudin5 For	CTCTGCTGGTTCGCCAACAT	(18)
GAPDH Rev	GAAGGTGAAGGTCGGAGTGAA	(18)
GAPDH For	TCCTGGAAGATGGTGATGGGA	(18)

REFERENCES AND NOTES

1. E. S. Cowley, I. Z. Chaves, F. Osman, G. Suen, K. Anantharaman, A. J. Hryckowian, Determinants of gastrointestinal group B *Streptococcus* carriage in adults. bioRxiv 553755 [Preprint] (2023). https://doi.org/10.110 1/2023.08.17.553755.

2. H. W. Wilkinson, Group B streptococcal infection in humans. Annu. Rev. Microbiol. 32, 41–57 (1978).

3. K. S. Doran, V. Nizet, Molecular pathogenesis of neonatal group B streptococcal infection: No longer in its infancy. Mol. Microbiol. 54, 23–31 (2004).

4. J. Hall, N. H. Adams, L. Bartlett, A. C. Seale, T. Lamagni, F. Bianchi-Jassir, J. E. Lawn, C. J. Baker, C. Cutland, P. T. Heath, M. Ip, K. L. Doare, S. A. Madhi, C. E. Rubens, S. K. Saha, S. Schrag, A. S.-T. Meulen, J. Vekemans, M. G. Gravett, Maternal disease with group B streptococcus and serotype distribution worldwide: Systematic review and metaanalyses.

Clin. Infect. Dis. 65, S112–S124 (2017).

5. A. Schuchat, Epidemiology of group B streptococcal disease in the United States: Shifting paradigms. Clin. Microbiol. Rev. 11, 497–513 (1998).

6. M. S. Edwards, M. A. Rench, A. A. Haffar, M. A. Murphy, M. M. Desmond, C. J. Baker, Long-term sequelae of group B streptococcal meningitis in infants. J. Pediatr. 106, 717–722 (1985).

7. GBD 2019 Antimicrobial Resistance Collaborators, Global mortality associated with 33 bacterial pathogens in 2019: A systematic analysis for the Global Burden of Disease Study 2019. Lancet 400, 2221–2248 (2022).

8. C. R. Phares, R. Lynfield, M. M. Farley, J. Mohle-Boetani, L. H. Harrison, S. Petit, A. S. Craig, W. Schaffner, S. M. Zansky, K. Gershman, K. R. Stefonek, B. A. Albanese, E. R. Zell, A. Schuchat, S. J. Schrag, Epidemiology of invasive group B streptococcal disease in the United States, 1999-2005. JAMA 299, 2056–2065 (2008).

9. A. Ohlsson, V. S. Shah, Intrapartum antibiotics for known maternal Group B streptococcal colonization. Cochrane Database Syst. Rev. 6, CD007467 (2014).

10. A. S. Romain, R. Cohen, C. Plainvert, C. Joubrel, S. Bechet, A. Perret, A. Tazi, C.

Poyart, C. Levy, Clinical and laboratory features of group B streptococcus meningitis in infants and newborns: Study of 848 cases in France, 2001-2014. Clin. Infect. Dis. 66, 857–864 (2018).
11. P. L. Graham III, P. Della-Latta, F. Wu, J. Zhou, L. Saiman, The gastrointestinal tract serves as the reservoir for Gram-negative pathogens in very low birth weight infants. Pediatr. Infect. Dis. J. 26, 1153–1156 (2007).
12. M. A. Carl, I. M. Ndao, A. C. Springman, S. D. Manning, J. R. Johnson, B. D. Johnston, C. A. Burnham, E. S. Weinstock, G. M. Weinstock, T. N. Wylie, M. Mitreva, S. Abubucker, Y. Zhou, H. J. Stevens, C. Hall-Moore, S. Julian, N. Shaikh, B. B. Warner, P. I. Tarr, Sepsis from the gut: The enteric habitat of bacteria that cause late-onset neonatal bloodstream infections. Clin. Infect. Dis. 58, 1211–1218 (2014).
13. A. Filleron, F. Lombard, A. Jacquot, E. Jumas-Bilak, M. Rodiere, G. Cambonie, H. Marchandin, Group B streptococci in milk and late neonatal infections: An analysis of cases in the literature. Arch. Dis. Child. Fetal Neonatal Ed. 99, F41–F47 (2014).
14. H. C. Dillon Jr., S. Khare, B. M. Gray, Group B streptococcal carriage and disease: A 6-year prospective study. J. Pediatr. 110, 31–36 (1987).
15. A. M. Weindling, J. M. Hawkins, M. A. Coombes, J. Stringer, Colonisation of babies and their families by group B streptococci. Br. Med. J. 283, 1503–1505 (1981).
16. L. Travier, M. Alonso, A. Andronico, L. Hafner, O. Disson, P. M. Lledo, S. Cauchemez, M. Lecuit, Neonatal susceptibility to meningitis results from the immaturity of epithelial barriers and gut microbiota. Cell Rep. 35, 109319 (2021).
17. G. O. Adeniyi-Ipadeola, J. D. Hankins, A. Kambal, X. L. Zeng, K. Patil, V. Poplaski, C. Bomidi, H. Nguyen-Phuc, S. L. Grimm, C. Coarfa, S. E. Crawford, S. E. Blutt, A. L. Speer, M. K. Estes, S. Ramani, Infant and adult human intestinal enteroids are morphologically and functionally distinct. bioRxiv 541350 [Preprint] (2023). https://doi.org/10.1101/2023.05.19.541350.
18. B. J. Kim, B. M. Hancock, A. Bermudez, N. Del Cid, E. Reyes, N. M. van Sorge, X. Lauth, C. A. Smurthwaite, B. J. Hilton, A. Stotland, A. Banerjee, J. Buchanan, R.

Wolkowicz, D. Traver, K. S. Doran, Bacterial induction of Snail1 contributes to bloodbrain barrier disruption. J. Clin. Invest. 125, 2473–2483 (2015).

19. K. Dominguez, A. K. Lindon, J. Gibbons, S. E. Darch, T. M. Randis, Group B Streptococcus drives major transcriptomic changes in the colonic epithelium. Infect. Immun. 91, e0003523 (2023).

20. M. J. Vaz, S. A. Purrier, M. Bonakdar, A. B. Chamby, A. J. Ratner, T. M. Randis, The impact of circulating antibody on group B *Streptococcus* intestinal colonization and invasive disease. Infect. Immun. 89, e00348-20 (2020).

21. M. J. Vaz, S. Dongas, A. J. Ratner, Capsule production promotes Group B Streptococcus intestinal colonization. Microbiol. Spectr. 11, e0234923 (2023).

22. L. R. Joyce, K. S. Doran, Gram-positive bacterial membrane lipids at the host-pathogen interface. PLOS Pathog. 19, e1011026 (2023).

23. L. R. Joyce, Z. Guan, K. L. Palmer, *Streptococcus pneumoniae, S. pyogenes and S. agalactiae* membrane phospholipid remodelling in response to human serum. Microbiology 167, 001048 (2021).

24. L. R. Joyce, H. S. Manzer, J. da C Mendonca, R. Villarreal, P. E. Nagao, K. S. Doran, K. L. Palmer, Z. Guan, Identification of a novel cationic glycolipid in *Streptococcus agalactiae* that contributes to brain entry and meningitis. PLoS Biol. 20, e3001555 (2022).

25. K. S. Doran, E. J. Engelson, A. Khosravi, H. C. Maisey, I. Fedtke, O. Equils, K. S. Michelsen, M. Arditi, A. Peschel, V. Nizet, Blood-brain barrier invasion by group B Streptococcus depends upon proper cell-surface anchoring of lipoteichoic acid. J. Clin. Invest. 115, 2499–2507 (2005).

26. A. Grundling, O. Schneewind, Genes required for glycolipid synthesis and lipoteichoic acid anchoring in *Staphylococcus aureus*. J. Bacteriol. 189, 2521–2530 (2007).

27. J. M. Kuypers, L. M. Heggen, C. E. Rubens, Molecular analysis of a region of the group B streptococcus chromosome involved in type III capsule expression. Infect. Immun. 57, 3058–3065 (1989).

28. H. W. Wilkinson, Nontypable group B streptococci isolated from human sources. J. Clin. Microbiol. 6, 183–184 (1977).
29. N. T. Reichmann, A. Grundling, Location, synthesis and function of glycolipids and polyglycerolphosphate lipoteichoic acid in Gram-positive bacteria of the phylum Firmicutes. FEMS Microbiol. Lett. 319, 97–105 (2011).
30. P. Henneke, S. Morath, S. Uematsu, S. Weichert, M. Pfitzenmaier, O. Takeuchi, A. Muller, C. Poyart, S. Akira, R. Berner, G. Teti, A. Geyer, T. Hartung, P. Trieu-Cuot, D. L. Kasper, D. T. Golenbock, Role of lipoteichoic acid in the phagocyte response to group B streptococcus. J. Immunol. 174, 6449–6455 (2005).
31. L. Deng, B. L. Spencer, J. A. Holmes, R. Mu, S. Rego, T. A. Weston, Y. Hu, G. F. Sanches, S. Yoon, N. Park, P. E. Nagao, H. F. Jenkinson, J. A. Thornton, K. S. Seo, A. H. Nobbs, K. S. Doran, The Group B Streptococcal surface antigen I/II protein, BspC, interacts with host vimentin to promote adherence to brain endothelium and inflammation during the pathogenesis of meningitis. PLOS Pathog. 15, e1007848 (2019).
32. B. L. Spencer, L. Deng, K. A. Patras, Z. M. Burcham, G. F. Sanches, P. E. Nagao, K. S. Doran, Cas9 contributes to group B streptococcal colonization and disease. Front. Microbiol. 10, 1930 (2019).
33. E. B. Andrade, J. Alves, P. Madureira, L. Oliveira, A. Ribeiro, A. Cordeiro-da-Silva, M. Correia-Neves, P. Trieu-Cuot, P. Ferreira, TLR2-induced IL-10 production impairs neutrophil recruitment to infected tissues during neonatal bacterial sepsis. J. Immunol. 191, 4759–4768 (2013).
34. S. D. Kobayashi, N. Malachowa, F. R. DeLeo, Neutrophils and bacterial immune evasion. J. Innate Immun. 10, 432–441 (2018).
35. B. Amulic, C. Cazalet, G. L. Hayes, K. D. Metzler, A. Zychlinsky, Neutrophil function: From mechanisms to disease. Annu. Rev. Immunol. 30, 459–489 (2012).
36. V. Nizet, T. Ohtake, X. Lauth, J. Trowbridge, J. Rudisill, R. A. Dorschner, V. Pestonjamasp, J. Piraino, K. Huttner, R. L. Gallo, Innate antimicrobial peptide protects the skin from invasive bacterial infection. Nature 414, 454–457 (2001).

37. R. Saar-Dover, A. Bitler, R. Nezer, L. Shmuel-Galia, A. Firon, E. Shimoni, P. Trieu-Cuot, Y. Shai, D-alanylation of lipoteichoic acids confers resistance to cationic peptides in group B streptococcus by increasing the cell wall density. PLOS Pathog. 8, e1002891 (2012).
38. M. Y. Kiriukhin, D. V. Debabov, D. L. Shinabarger, F. C. Neuhaus, Biosynthesis of the glycolipid anchor in lipoteichoic acid of *Staphylococcus aureus* RN4220: Role of YpfP, the diglucosyldiacylglycerol synthase. J. Bacteriol. 183, 3506–3514 (2001).
39. C. Theilacker, P. Sanchez-Carballo, I. Toma, F. Fabretti, I. Sava, A. Kropec, O. Holst, J. Huebner, Glycolipids are involved in biofilm accumulation and prolonged bacteraemia in *Enterococcus faecalis*. Mol. Microbiol. 71, 1055–1069 (2009).
40. R. Bals, X. Wang, M. Zasloff, J. M. Wilson, The peptide antibiotic LL-37/hCAP-18 is expressed in epithelia of the human lung where it has broad antimicrobial activity at the airway surface. Proc. Natl. Acad. Sci. U.S.A. 95, 9541–9546 (1998).
41. E. D. Stolzenberg, G. M. Anderson, M. R. Ackermann, R. H. Whitlock, M. Zasloff, Epithelial antibiotic induced in states of disease. Proc. Natl. Acad. Sci. U.S.A. 94, 8686–8690 (1997).
42. S. M. Lawrence, R. Corriden, V. Nizet, Age-appropriate functions and dysfunctions of the neonatal neutrophil. Front. Pediatr. 5, 23 (2017).
43. A. A. Navarini, K. S. Lang, A. Verschoor, M. Recher, A. S. Zinkernagel, V. Nizet, B. Odermatt, H. Hengartner, R. M. Zinkernagel, Innate immune-induced depletion of bone marrow neutrophils aggravates systemic bacterial infections. Proc. Natl. Acad. Sci. U.S.A. 106, 7107–7112 (2009).
44. G. C. W. Bee, K. L. Lokken-Toyli, S. T. Yeung, L. Rodriguez, T. Zangari, E. E. Anderson, S. Ghosh, C. V. Rothlin, P. Brodin, K. M. Khanna, J. N. Weiser, Agedependent differences in efferocytosis determine the outcome of opsonophagocytic protection from invasive pathogens. Immunity 56, 1255–1268.e5 (2023).
45. R. D. Christensen, T. E. Harper, G. Rothstein, Granulocyte-macrophage progenitor cells in term and preterm neonates. J. Pediatr. 109, 1047–1051 (1986).

46. O. Levy, S. Martin, E. Eichenwald, T. Ganz, E. Valore, S. F. Carroll, K. Lee, D. Goldmann, G. M. Thorne, Impaired innate immunity in the newborn: Newborn neutrophils are deficient in bactericidal/permeability-increasing protein. Pediatrics 104, 1327–1333 (1999).

47. D. C. Anderson, O. Abbassi, T. K. Kishimoto, J. M. Koenig, L. V. McIntire, C. W. Smith, Diminished lectin-, epidermal growth factor-, complement binding domain-cell adhesion molecule-1 on neonatal neutrophils underlies their impaired CD18-independent adhesion to endothelial cells in vitro. J. Immunol. 146, 3372–3379 (1991).

48. S. A. Kristian, V. Datta, C. Weidenmaier, R. Kansal, I. Fedtke, A. Peschel, R. L. Gallo, V. Nizet, D-alanylation of teichoic acids promotes group a streptococcus antimicrobial peptide resistance, neutrophil survival, and epithelial cell invasion. J. Bacteriol. 187, 6719–6725 (2005).

49. N. Fisher, L. Shetron-Rama, A. Herring-Palmer, B. Heffernan, N. Bergman, P. Hanna, The *dltABCD* operon of *Bacillus anthracis* sterne is required for virulence and resistance to peptide, enzymatic, and cellular mediators of innate immunity. J. Bacteriol. 188, 1301–1309 (2006).

50. A. Peschel, R. W. Jack, M. Otto, L. V. Collins, P. Staubitz, G. Nicholson, H. Kalbacher, W. F. Nieuwenhuizen, G. Jung, A. Tarkowski, K. P. van Kessel, J. A. van Strijp, Staphylococcus aureus resistance to human defensins and evasion of neutrophil killing via the novel virulence factor MprF is based on modification of membrane lipids with llysine. J. Exp. Med. 193, 1067–1076 (2001).

51. C. N. LaRock, V. Nizet, Cationic antimicrobial peptide resistance mechanisms of streptococcal pathogens. Biochim. Biophys. Acta 1848, 3047–3054 (2015).

52. H. S. Joo, M. Otto, Mechanisms of resistance to antimicrobial peptides in staphylococci. Biochim. Biophys. Acta 1848, 3055–3061 (2015).

53. C. Poyart, M. C. Lamy, C. Boumaila, F. Fiedler, P. Trieu-Cuot, Regulation of D-alanyllipoteichoic acid biosynthesis in Streptococcus agalactiae involves a novel twocomponent regulatory system. J. Bacteriol. 183, 6324–6334 (2001).

54. C. Poyart, E. Pellegrini, M. Marceau, M. Baptista, F. Jaubert, M. C. Lamy, P. Trieu-Cuot, Attenuated virulence of *Streptococcus agalactiae* deficient in D-alanyl-lipoteichoic acid is due to an increased susceptibility to defensins and phagocytic cells. Mol. Microbiol. 49, 1615–1625 (2003).
55. Y. Ramos, S. Sansone, S. M. Hwang, T. A. Sandoval, M. Zhu, G. Zhang, J. R. Cubillos-Ruiz, D. K. Morales, Remodeling of the enterococcal cell envelope during surface penetration promotes intrinsic resistance to stress. MBio 13, e0229422 (2022).
56. S. Lehnardt, P. Henneke, E. Lien, D. L. Kasper, J. J. Volpe, I. Bechmann, R. Nitsch, J. R. Weber, D. T. Golenbock, T. Vartanian, A mechanism for neurodegeneration induced by group B streptococci through activation of the TLR2/MyD88 pathway in microglia. J. Immunol. 177, 583–592 (2006).
57. J. C. Chang, J. C. Jimenez, M. J. Federle, Induction of a quorum sensing pathway by environmental signals enhances group A streptococcal resistance to lysozyme. Mol. Microbiol. 97, 1097–1113 (2015).
58. T. A. Hooven, M. Bonakdar, A. B. Chamby, A. J. Ratner, A counterselectable sucrose sensitivity marker permits efficient and flexible mutagenesis in *Streptococcus agalactiae*. Appl. Environ. Microbiol. 85, e03009-18 (2019).
59. A. Jeng, V. Sakota, Z. Li, V. Datta, B. Beall, V. Nizet, Molecular genetic analysis of a group A Streptococcus operon encoding serum opacity factor and a novel fibronectinbinding protein, SfbX. J. Bacteriol. 185, 1208–1217 (2003).
60. L. R. Joyce, Z. Guan, K. L. Palmer, Phosphatidylcholine biosynthesis in mitis group streptococci via host metabolite scavenging. J. Bacteriol. 201, e00495-19 (2019).
61. H. M. Adams, L. R. Joyce, Z. Guan, R. L. Akins, K. L. Palmer, Streptococcus mitis and S. oralis lack a requirement for CdsA, the enzyme required for synthesis of major membrane phospholipids in bacteria. Antimicrob. Agents Chemother. 61, e02552-16 (2017).
62. K. A. Geno, J. S. Saad, M. H. Nahm, Discovery of novel pneumococcal serotype 35D, a natural WciG-deficient variant of serotype 35B. J. Clin. Microbiol. 55, 1416–1425 (2017).

63. J. Derk, C. N. Como, H. E. Jones, L. R. Joyce, S. Kim, B. L. Spencer, S. Bonney, R. O'Rourke, B. Pawlikowski, K. S. Doran, J. A. Siegenthaler, Formation and function of the meningeal arachnoid barrier around the developing mouse brain. Dev. Cell 58, 635–644.e4 (2023).
64. L. Thomer, C. Emolo, V. Thammavongsa, H. K. Kim, M. E. McAdow, W. Yu, M. Kieffer, O. Schneewind, D. Missiakas, Antibodies against a secreted product of Staphylococcus aureus trigger phagocytic killing. J. Exp. Med. 213, 293–301 (2016).
65. A. L. Dumont, T. K. Nygaard, R. L. Watkins, A. Smith, L. Kozhaya, B. N. Kreiswirth, B. Shopsin, D. Unutmaz, J. M. Voyich, V. J. Torres, Characterization of a new cytotoxin that contributes to Staphylococcus aureus pathogenesis. Mol. Microbiol. 79, 814–825 (2011).
66. R. L. Burton, M. H. Nahm, Development and validation of a fourfold multiplexed opsonization assay (MOPA4) for pneumococcal antibodies. Clin. Vaccine Immunol. 13, 1004–1009 (2006).
67. E. T. Berends, A. R. Horswill, N. M. Haste, M. Monestier, V. Nizet, M. von Kockritz-Blickwede, Nuclease expression by Staphylococcus aureus facilitates escape from neutrophil extracellular traps. J. Innate Immun. 2, 576–586 (2010).
68. A. M. Brady, J. J. Calix, J. Yu, K. A. Geno, G. R. Cutter, M. H. Nahm, Low invasiveness of pneumococcal serotype 11A is linked to ficolin-2 recognition of O-acetylated capsule epitopes and lectin complement pathway activation. J Infect. Dis. 210, 1155–1165 (2014).
69. H. H. Yim, A. Nittayarin, C. E. Rubens, Analysis of the capsule synthesis locus, a virulence factor in group B streptococci. Adv. Exp. Med. Biol. 418, 995–997 (1997).
70. C. Faralla, M. M. Metruccio, M. De Chiara, R. Mu, K. A. Patras, A. Muzzi, G. Grandi, I. Margarit, K. S. Doran, R. Janulczyk, Analysis of two-component systems in group B Streptococcus shows that RgfAC and the novel FspSR modulate virulence and bacterial fitness. MBio 5, e00870-14 (2014).
71. B. L. Spencer, A. Chatterjee, B. A. Duerkop, C. J. Baker, K. S. Doran, Complete genome sequence of neonatal clinical group B streptococcal isolate CJB111. Microbiol. Resour. Announc. 10, e01268-20 (2021).

72. M. J. Casadaban, S. N. Cohen, Analysis of gene control signals by DNA fusion and cloning in Escherichia coli. J. Mol. Biol. 138, 179–207 (1980).
